# Multicentre Harmonisation of a Six-Colour Flow Cytometry Panel for Naïve/Memory T Cell Immunomonitoring

**DOI:** 10.1155/2020/1938704

**Published:** 2020-04-12

**Authors:** Iole Macchia, Valentina La Sorsa, Irene Ruspantini, Massimo Sanchez, Valentina Tirelli, Maria Carollo, Giorgio Fedele, Pasqualina Leone, Giovanna Schiavoni, Carla Buccione, Paola Rizza, Paola Nisticò, Belinda Palermo, Stefania Morrone, Helena Stabile, Aurelia Rughetti, Marianna Nuti, Ilaria Grazia Zizzari, Cinzia Fionda, Roberta Maggio, Cristina Capuano, Concetta Quintarelli, Matilde Sinibaldi, Chiara Agrati, Rita Casetti, Andrea Rozo Gonzalez, Floriana Iacobone, Angela Gismondi, Filippo Belardelli, Mauro Biffoni, Francesca Urbani

**Affiliations:** ^1^Department of Oncology and Molecular Medicine, Istituto Superiore di Sanità (ISS), Rome 00161, Italy; ^2^Research Coordination and Support Service, CoRI, ISS, Rome 00161, Italy; ^3^Core Facilities, ISS, Rome 00161, Italy; ^4^Department of Infectious Diseases, ISS, Rome 00161, Italy; ^5^Center for Gender-Specific Medicine, ISS, Rome 00161, Italy; ^6^Unit of Tumor Immunology and Immunotherapy, IRCCS Regina Elena National Cancer Institute (IRE), Rome 00128, Italy; ^7^Department of Experimental Medicine, Sapienza University of Rome (SUR), 00185, Italy; ^8^Department of Molecular Medicine, SUR, Rome 00185, Italy; ^9^Clinical Research, Imperial College, London W12 ONN, UK; ^10^Onco-Hematology Department, IRCCS Bambino Gesù Children's Hospital (OPBG), Rome 00165, Italy; ^11^Cellular Immunology Laboratory, IRCCS National Institute for Infectious Diseases “L. Spallanzani” (INMI), Rome 00149, Italy; ^12^Institute of Translational Pharmacology, National Research Council, Rome 00185, Italy; ^13^Medical Biotechnology and Translational Medicine PhD School, Tor Vergata University, Rome 00133, Italy

## Abstract

**Background:**

Personalised medicine in oncology needs standardised immunological assays. Flow cytometry (FCM) methods represent an essential tool for immunomonitoring, and their harmonisation is crucial to obtain comparable data in multicentre clinical trials. The objective of this study was to design a harmonisation workflow able to address the most effective issues contributing to intra- and interoperator variabilities in a multicentre project.

**Methods:**

The Italian National Institute of Health (Istituto Superiore di Sanità, ISS) managed a multiparametric flow cytometric panel harmonisation among thirteen operators belonging to five clinical and research centres of Lazio region (Italy). The panel was based on a backbone mixture of dried antibodies (anti-CD3, anti-CD4, anti-CD8, anti-CD45RA, and anti-CCR7) to detect naïve/memory T cells, recognised as potential prognostic/predictive immunological biomarkers in cancer immunotherapies. The coordinating centre distributed frozen peripheral blood mononuclear cells (PBMCs) and fresh whole blood (WB) samples from healthy donors, reagents, and Standard Operating Procedures (SOPs) to participants who performed experiments by their own equipment, in order to mimic a real-life scenario. Operators returned raw and locally analysed data to ISS for central analysis and statistical elaboration.

**Results:**

Harmonised and reproducible results were obtained by sharing experimental set-up and procedures along with centralising data analysis, leading to a reduction of cross-centre variability for naïve/memory subset frequencies particularly in the whole blood setting.

**Conclusion:**

Our experimental and analytical working process proved to be suitable for the harmonisation of FCM assays in a multicentre setting, where high-quality data are required to evaluate potential immunological markers, which may contribute to select better therapeutic options.

## 1. Introduction

Immunomonitoring has become increasingly relevant in the immunooncology field for the identification of potential prognostic/predictive immune biomarkers and a better understanding of their underlying mechanisms of action, leading to improved personalised treatments.

FCM is a powerful technology commonly used to dissect the immune phenotype due to its unique ability to measure quantitatively and simultaneously large numbers of single cells by multiple markers. However, given the complexity of polychromatic assays currently used in immunotherapy studies, a harmonisation process is crucial to guarantee reproducibility of data among laboratories, especially in multicentre trials [1–3].

In most cases, monitoring of patients' response is conducted over an extended time lapse, from the pretreatment phase to the follow-up period, and in this context, assay repeatability must be considered mandatory. For this reason, FCM assays need to meet specific analytical method validation especially in settings requiring good laboratory/clinical practice (GLP/GCP) compliance. According to the Food and Drug Administration (FDA), validation is realised to confirm that the method is fit for its intended use (“fit-for-purpose, FFP”), satisfying specific criteria with respect to stability, accuracy, precision, specificity, detection limit, linearity, ruggedness, and robustness [4–7]. Nevertheless, continuous improvement of best practices and evolution of immune cell phenotyping requires periodic upgrading of FCM-based methods and technology [8].

Currently, the only existing guidelines for validating flow cytometric assays are gathered from guidance documents and recommendations published in specific journals [9, 10]. So far, several harmonisation and standardisation programs have been promoted and activated, e.g., by the Human Immune Phenotyping Consortium (HIPC) [11], the EuroFlow Consortium [3, 12], and the ONE Study Consortium [13].

In clinical cancer studies, due to the feasibility and reproducibility of blood compared to tumour tissue sampling, immune phenotyping of peripheral blood leukocyte subpopulations is considered as a valuable approach to explore systemic immunological markers that can facilitate patient selection and treatment decisions [14]. In particular, several papers reported clinical significance of naïve/memory T cell subsets as potential biomarkers with a *prognostic* and/or *predictive of response to immunotherapy* potential, in different cancer types, such as non-small cell lung cancer (NSCLC) [15] and melanoma [16, 17]. Four functional T cell compartments can be defined in humans by the expression of CC-chemokine receptor 7 (CCR7) and CD45RA: naïve (N, CCR7+CD45RA+); central memory (CM, CCR7+CD45RA−); effector memory (EM, CCR7−CD45RA−), and terminally differentiated effector (TD, CCR7−CD45RA+) T cells [18, 19].

In this study, we present results of a harmonisation across laboratories belonging to the “Lazio Network for Translational Medicine and Tumour Biotherapies” of a six-colour FCM panel, designed for the identification of naïve/memory T cell subsets in human cryopreserved PBMCs (cPBMCs) or WB samples. Our workflow has been conceived to address the most effective issues contributing to intra- and interoperator variability and the impact of analysis centralisation on global results, according to a scheme analogous to a proficiency testing program. To this aim, the ISS reference centre managed logistics and distributed cPBMC and fresh WB samples, as well as reagents and SOPs, to thirteen operators distributed in five centres. Each participant performed experiments in replicates by his own equipment and sent raw and locally analysed data to ISS, which evaluated variability of both local and central analyses as well as operator/laboratory performance. The whole process highlighted critical aspects of a harmonisation process and emphasised advantages of sharing dried reagents and detailed SOPs as well as of centralising the preanalytical phase and data analysis. Overall, our data represent a real-life example of harmonisation workflow to dissect and manage variability related to FCM immune-monitoring assays, especially in multicentre clinical studies.

## 2. Methods

### 2.1. Survey

As a working unit of the “Lazio regional network for translational medicine” (Italy), we started our activity by surveying clinical and research groups of Lazio region involved in immunomonitoring. A questionnaire (supplementary material ([Supplementary-material supplementary-material-1])) was distributed to explore their expertise, equipment, and interest in joining a harmonisation process for immunophenotyping of human leukocyte subsets. Five institutions (8 different laboratories, for a total number of 13 operators, including a reference operator, ROP), from different fields and organizations, positively answered to the survey and adhered to the project: ISS, SUR, IRE, OPBG, and INMI. Each operator was assigned a confidential ID (from Op_A to Op_M) only known by the operator himself and by the ISS coordinator group. A complete list of participants, instruments, and software is reported in Table 1.

### 2.2. Instrument Characteristics

Seven different instruments with compatible optical configuration (three flow cytometer models: Beckman Coulter (BC, Miami, FL); Gallios™, Becton Dickinson (BD, San Jose, CA); FACSCanto™ II; and BD LSRFortessa™) were used (Table 1). All the instruments had to fulfil minimum requirements such as to support two laser sources (488 and 633/640 nm) and to undergo an internal quality control of alignment (required), sensitivity, and linearity (highly recommended). ISS BD FACSCanto™ I+ underwent a fluidic system upgrade (therefore comparable to BD FACSCanto™ II), and it was the only instrument of the project maintained according to the GMP standard of quality assurance; all other cytometers were dedicated to research use only. All instruments were equipped with the manufacturer's default optical configuration and were calibrated by using the BD FACSDiva™ Cytometer Setting and Tracking System Beads, except BC Gallios (BC Flow-Check Fluorospheres and Spherotech, Lake Forest, IL; Rainbow Calibration Particles, 8 peaks).

### 2.3. Experimental Workflow

**Figure 1:**
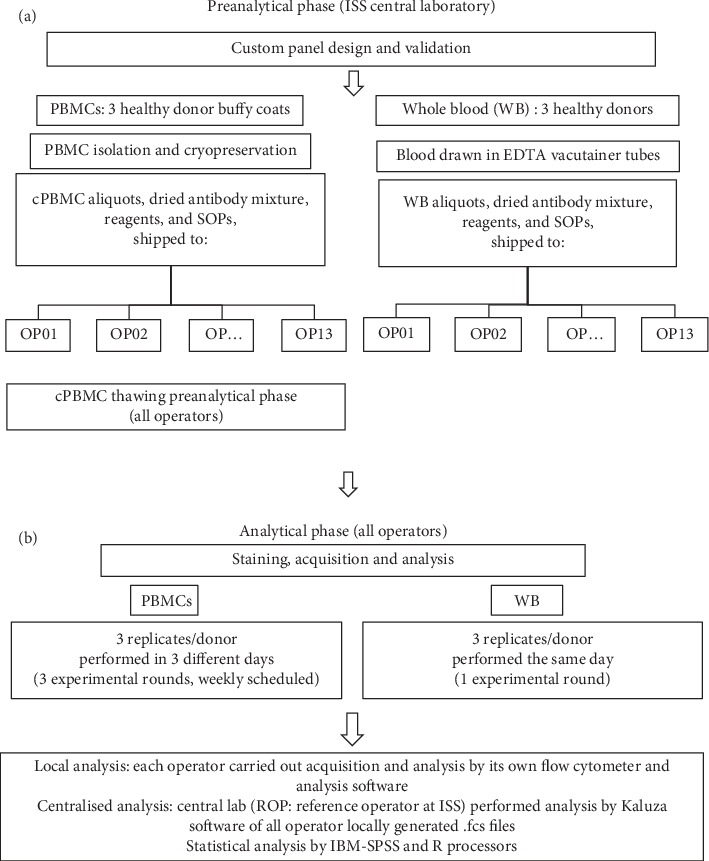
Flowchart. Workflow of the multicentre harmonisation of a six-colour flow cytometry panel for naïve/memory T cell immunomonitoring.

The experimental design shown in Figure 1 describes preanalytical and analytical phases.

#### 2.3.1. Preanalytical Phase

The ISS main centre defined a naïve/memory T cell panel, by selecting monoclonal antibody clones and antibody-fluorochrome combinations, in accordance with FCM panel design rules [20]. Each selected monoclonal antibody in liquid formulation was previously titrated to achieve the highest resolution index [21]. We licensed the supplier for the production of customised dried antibody tubes after a prevalidation test carried out on five healthy cPBMC donors and a stability testing assay conducted on a WB sample, in 8 replicates for each time point (data not shown). The proposed staining cocktail was a six-colour custom reagent, based on the *backbone and drop-in* concept, composed by a mixture of five dried antibodies, essential for naïve/memory identification [19] (CD4-FITC, CCR7-PE, CD8-PeCy5.5, CD3-PeCy7, and CD45RA-APC), produced by the DURAClone Technology (Beckman Coulter, #B38658, Life Science Europe, Geneva, Swiss); the dried mixture was integrated with a live/dead discriminator (LIVE/DEAD™ Fixable Near-IR Dead Cell Stain Kit, Thermo Fisher Scientific, Waltham, MA) for (a) cPBMCs or with CD45 APC-Cy7 (BioLegend, San Diego, CA) in liquid format for (b) WB staining (Table 2).

Reagents and SOPs containing experimental procedures and including guidelines for gating strategy were provided to peripheral sites that stained in triplicate two blood sample sets: (i) cPBMCs isolated from buffy coats (*n* = 3); (ii) WB samples (*n* = 3). The number of replicates was chosen based on previous reports [9]. For logistic reasons, buffy coat and WB samples were drawn from different groups of healthy donors, collected in two separate days (Figure 1(a)).

- *cPBMC Isolation, Cryopreservation, and Delivery*. This phase was centralised to avoid variability related to PBMC isolation and freezing procedures (detailed in supplementary material). PBMCs were isolated from three healthy donor buffy coats (named PBMC1, PBMC2, and PBMC3) collected at the Policlinico Umberto I blood bank that usually releases buffy coat bags 24 hours after blood collection as a safety policy. cPBMC samples (4 vials per donor), as well as DURAClone tubes, single-colour DURAClone compensation tubes, and one stock of LIVE/DEAD Stain Kit, were shipped to participating centres. cPBMC vials were delivered in dry ice and stored in liquid nitrogen upon arrival at peripheral sites.
- *WB Sample Collection and Delivery*. Aliquots of WB drawn by three healthy donors (named WB1, WB2, and WB3), collected in sodium-EDTA Vacutainer (BD) tubes, were sent to the participants within the same day, along with DURAClone tubes, one set of single-colour DURAClone tubes for compensation, and one stock of anti-CD45 APC-Cy7, and were maintained at room temperature until staining (24 h later).

#### 2.3.2. Analytical Phase

Each operator received reagents and detailed instructions for instrument set-up, sample staining, acquisition, and data analysis (Figure 1(b)), specified in commonly agreed SOPs (detailed in the supplementary material).

- *cPBMC Thawing and Staining*. The participants were asked to determine viability after cell thawing by means of trypan-blue exclusion cell count according to the “cPBMC thawing and counting” SOP. Afterwards, cPBMCs were stained in three separate days (three experimental rounds for each donor), at weekly scheduled intervals, according to the “PBMC staining, acquisition, and analysis” SOP.
- *WB Staining*. Staining was performed in triplicate for each donor 24 hours later from blood collection, according to “WB staining, acquisition, and analysis” SOP. Cells were fixed and acquired the day after.

#### 2.3.3. Flow Cytometry Local and Central Analysis: Gating Strategy

**Figure 2:**
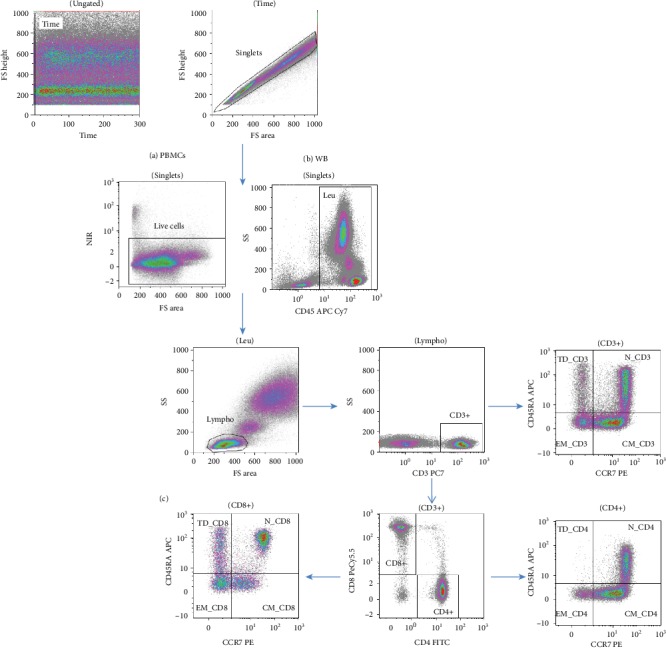
Gating strategy. Representative dot plots of cPBMC (a) and WB (b) samples: after selecting the window of instrumental stable acquisition time lapse and singlet events, lymphocytes were gated within live cells (a) or CD45+ cells/leukocytes (b). CD3+ cells were subsequently identified within the lymphocyte gate (common gate for (a) and (b) analyses) and further distinguished in CD4+ and CD8+ lymphocytes. After that, naïve/memory subsets were identified within CD3+, CD4+, and CD8+ T cells through CD45RA/CCR7 combination (c).

Gating hierarchy was included in both cPBMC and WB staining SOPs. Exemplary gating strategy is shown in Figure 2, which depicts sequential steps for selecting the population of interest, up to the identification of naïve/memory T cell subsets. Briefly, for both cPBMC and WB samples, we first selected the window of instrumental stable acquisition time lapse and singlet events. Lymphocytes were then gated within live cells for cPBMCs (Figure 2(a)) or within the CD45+ leukocyte region for WB (Figure 2(b)), and CD3+ cells were subsequently identified within the lymphocyte gate. Furthermore, participants were asked to draw a CD4 vs. CD8 expression plot, within CD3+ T cells, to gate single-positive events, excluding CD4+CD8+ double-positive T cells, which represent a functionally distinct cell subset with peculiar behaviour, as described in several infectious and oncologic diseases [22, 23]. Cells were further distinguished in naïve/memory subsets through the CD45RA/CCR7 combination (Figure 2(c)), in CD3+, CD4+, and CD8+ events. Concerning FCM analysis, the guidelines recommended to properly adjust biexponential scaling to better visualise a specific cell population [24]. Data sets underwent two different postacquisition analyses: (i) in the local analysis, each participant individually applied compensation and gating strategies using his/her own analysis software, reported percentages of defined cell subsets on a shared register, and sent it back to the ISS main centre (Supplementary [Supplementary-material supplementary-material-1]); (ii) in the central analysis, an expert flow cytometry “reference operator” belonging to the ISS coordinating laboratory, defined as ROP (Op_M), reanalysed all local raw fcs files using Kaluza software.

#### 2.3.4. Data Analysis

Data analysis was carried out using IBM-SPSS V25 and R V3.6.1 software [25].

Operator performance was assessed by means of two different indicators:

- *Z-Score*. This indicator measured how many standard deviations a participant observation differs from that of ROP. For each blood sample type, donor, and parameter, the *Z*-score was calculated as *Z* = (*X*–*μ*)/*σ*, where *X* is the operator observation (average of donor-specific triplicate), *μ* is the average, and *σ* is the standard deviation of *n* = 12 values measured by ROP in a centralised analysis. Then, the median of the donor-related values was calculated. We classified the scores as follows: satisfactory if ∣*Z*‐score∣ < 2.0, questionable if 2.0 ≤ ∣*Z*‐score∣ < 3.0, and unsatisfactory if ∣*Z*‐score∣ ≥ 3
- *Coefficient of Variation (CV* = *SD*/*Mean*). Intraoperator precision in specific parameter analysis is shown as the median value of CVs estimated on each donor triplicate. We generally considered acceptable CV values below 0.20.

Interoperator variability was estimated by means of

- *CV*. This was calculated for each parameter as the median of operator CVs, derived from the average donor-triplicate values. Individual CV was used to estimate repeatability in intra/interassay analysis of each operator observations when evaluating a donor sample in three replicates
- *Bias*. This was estimated as an expression of operator closeness to the reference value. The reference value, determined as the average of the values obtained by all the operators, centrally analysed by the ROP for each parameter. For each participant, donor, parameter, and blood sample type, bias was assessed as the difference between the average of triplicate values measured by the participant and the reference value, then divided by the reference value. The absolute value of bias was then determined, and the median value of operators' bias for each parameter and blood type is shown. For bias, we generally considered as acceptable values below 0.20
- *Intraclass Correlation (ICC)*. Data reproducibility was determined for each cell subset as the agreement among analysts in measuring the same subject. To this aim, we used ICC, a statistical index independent of marker population size that estimates the ratio of biological (due to differences in donors) to total variability, as previously described [26, 27], and calculated in a 2-way design with “analyst” and “donor” as random variables for each parameter, considering the mean value of donor triplicates. Acceptability threshold was defined by ICC > 0.75

Multivariate analysis was performed using PCA as implemented in the built-in R function prcomp(), a dimension-reduction tool that can be used to reduce a set of variables to a smaller set that still contains most of the information, as explained variance, in the original set. Data were first centred and scaled for each donor and sample type. Parent populations and subpopulation were analysed separately. Biplots were drawn using the “ggbiplot” R package.

### 2.4. Ethical Issues

This study was coordinated by ISS (Rome, Italy). All experimental protocols were approved by ISS and all methods were carried out in accordance with the Declaration of Helsinki and other relevant international guidelines [28]. Blood samples were collected from healthy donors enrolled at Policlinico Umberto I blood bank (Rome, Italy) after obtaining their written informed consent.

## 3. Results

To harmonise our naïve/memory panel, we evaluated variability of data sets obtained from thirteen experienced operators working in five independent centres of the Lazio region, using seven different instruments (three flow cytometers models) equipped with compatible laser and detector/filter settings and three different software of analysis (Table 1).

In two out of the five centres, multiple operators participated to the harmonisation panel, some of them working on different flow cytometers and using different analysis software. Therefore, hereafter, we will refer intra- and interoperator variability as expression of the contribution of each operator independently from the centre. cPBMC replicates were obtained in different experimental rounds (interassay replicates), whereas WB replicates were carried out within the same day (intra-assay), as previously stated.

### 3.1. cPBMC Recovery and Viability

Results on recovery and viability of cPBMCs after thawing revealed that this experimental phase was not subjected to large variability among operators as most of them (10/13) showed a coefficient of variation (CV) between 0.06 and 0.20 for the recovery index and 12/13 showed CV value below 0.11 for the viability index (Supplementary Fig. [Supplementary-material supplementary-material-1]). Regarding WB, six of the total processed samples (*n* = 117) were excluded from the graphic and statistical analysis (outliers) due to problems related to red blood cell lysis and other artefacts, as reported by participants.

### 3.2. Distribution of T Cell Populations

**Figure 3:**
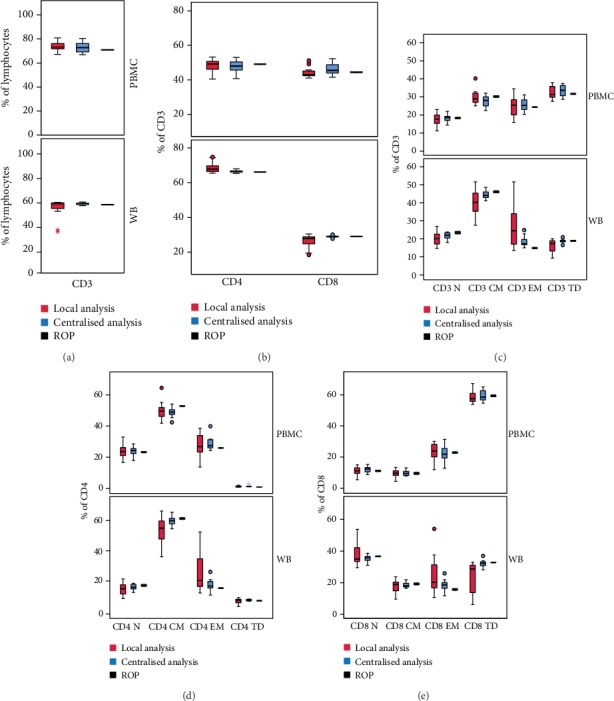
Distribution of T cell populations. Box plots represent percentages (%) of (a) CD3 (gated within lymphocytes); (b) CD4 and CD8 (gated within CD3 cells); naïve/memory T cell subsets gated within (c) CD3, (d) CD4, and (e) CD8 cells. Cell frequencies obtained by all operators, from one representative cPBMC (upper panels) and one representative WB sample (lower panels), are shown. In yellow boxes, percentages obtained by the ROP are reported.

We compared local vs. centralised analysis of all T cell subset frequencies by mean of boxes representing triplicate mean values obtained in all observations (*n* = 12), from one representative donor for cPBMC (upper box-plot panel) and one for WB samples (lower box-plot panel, Figure 3). In yellow boxes, percentages obtained by ROP are reported.

As expected, major CD3+ (gated within lymphocytes; Figure 3(a)), CD4+, and CD8+ (gated within CD3+ cells; Figure 3(b)) populations showed a very limited dispersion of frequencies if compared to naïve/memory subsets (Figures 3(c)–3(e)), especially in the central analysis. Dispersion of frequency profiles was far more evident in locally analysed WB as compared to cPBMC samples. However, centralisation reduced it to a greater extent in WB setting.

Of note, regarding cPBMC plots, we found a CD4/CD8 ratio lower than the normal expected value and a very high percentage of TD CD8 T cells with a consequently altered relative proportion of the other memory subsets (Figure 3(b)). ROP results (yellow boxes) were mostly comparable to all operator median values.

### 3.3. Intraoperator Variability/Operator Performance

The number of participants was suitable to assess operator consistency to SOP through a proficiency testing-like approach.

**Figure 4:**
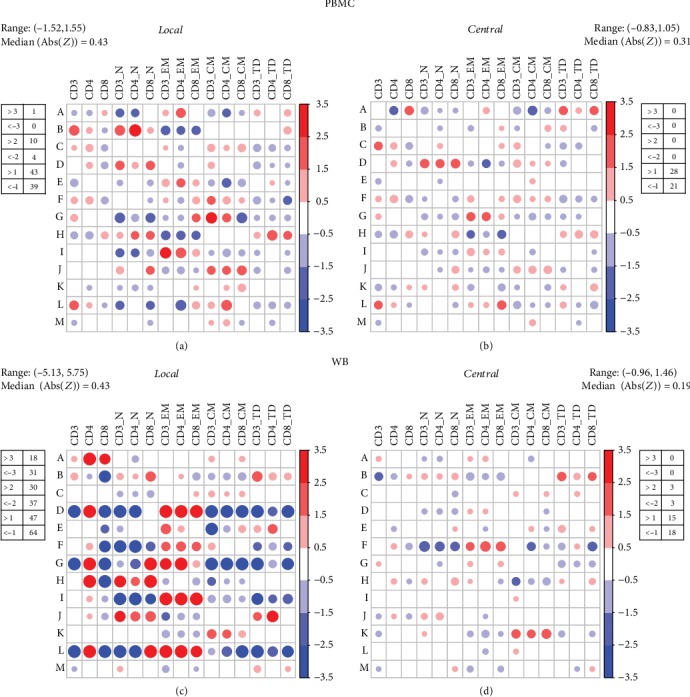
Operator performance. *Z*-score measured how much the operators overestimate (red) or underestimate (blue) the values with respect to an average reference value (average of the values obtained from all the operators centrally analysed by the ROP). Heat maps represent a detailed study of concordance with the average reference results, relative to each separate parameter for cPBMC (a, b) and WB (c, d) locally (a, c) and centrally (b, d) analysed.

To address the performance of all participants, we evaluated intraoperator repeatability (i.e., variability of individual measurements on the same donor, expressed as CV in Supplementary Fig. [Supplementary-material supplementary-material-1]), as well as standardised residuals (i.e., *Z*-score; Figure 4) [29], on both cPBMC and WB specimens, for each analysed parameter.

Concerning repeatability, interassay analysis in cPBMC globally revealed a maximum CV value of 1.32 vs. 0.75 and median CV values of 0.13 vs. 0.14 in local vs. central results (Supplementary Fig. [Supplementary-material supplementary-material-1] and [Supplementary-material supplementary-material-1]). However, in this setting, CVs for many of the parameters are often laid above the acceptability threshold (52/195 and 56/195, by local and central analysis, respectively). Of note, for some operators, such as Op_G, centralisation reduced the CV values up to levels that indeed were still not acceptable (Supplementary Fig. [Supplementary-material supplementary-material-1] and [Supplementary-material supplementary-material-1] and Figures 4(a) and 4(b)). On the other hand, in WB intra-assay analysis, CV values were excellent (by far below 0.20) for almost all operators (only 5/195 and 2/195 above the 0.20 threshold), with maximum CV values of 0.28 and 0.29, respectively, and a median CV value of 0.03 for both local and central analyses (Supplementary Fig. [Supplementary-material supplementary-material-1] and [Supplementary-material supplementary-material-1]). The lower operator precision observed in cPBMCs in terms of CV can be mainly ascribable to the complex preanalytical phase in this setting (thawing).

In central analysis, *Z*-score was never found unsatisfactory (Figures 4(b) and 4(d)), while in local analysis, some parameters were under- or overestimated, slightly in cPBMC (1/195) and notably in WB (49/195) setting (Figures 4(a) and 4(c), respectively). Therefore, we can argue that centralisation marginally influenced operator performance in cPBMCs, while it considerably impacted on WB setting, suggesting that local WB variability, for certain operators, was mostly affected by analysis type, rather than staining and acquisition phase.

Of note, *ex post* analysis of reference operator (ROP: Op_M) performance revealed that this operator reached one of the best scores among all operators.

### 3.4. Interoperator Variability/Subpopulation Reliability

**Figure 5:**
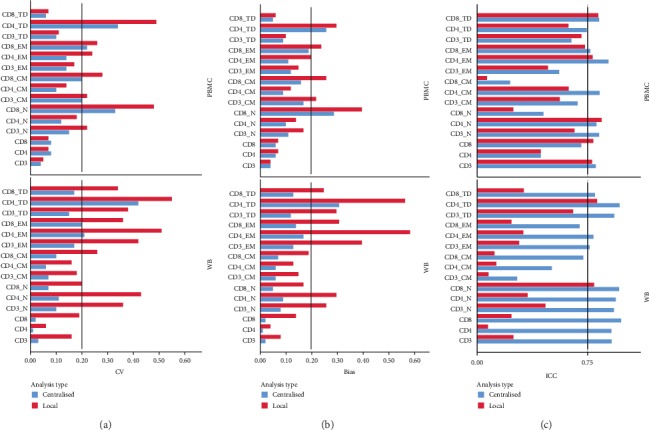
Interoperator variability. Overall interoperator variability related to the identification of CD3, CD4, and CD8 major subpopulations and naïve/memory T cell subsets, by all operators, in cPBMC (upper panel) and WB (lower panel) samples. Interoperator variance was expressed as (a) CV calculated as the median of analyst-specific CV, derived from the three average donor triplicates for each marker; (b) bias, calculated with respect to the mean reference value (average of the values obtained from all the operator data centrally analysed by the ROP); and (c) ICC, as an index of reproducibility, calculated in a 2-way design with “analyst” and “donor” as random variables for each parameter, considering the mean value of donor triplicates. CV, bias, and ICC among sites are shown for local (red bar) and centralised (blue bar) data analyses.

In order to address the impact of analysis type (centralised vs. local) on overall interoperator measurement uncertainty, we evaluated each parameter on both the cPBMC and WB sets of experiments: (a) reproducibility expressed as CV, (b) bias calculated with respect to the reference value, and (c) agreement by means of ICC (Figure 5).

In Figure 5(a), bars represent median values among the three donor-specific CVs (obtained on the three replicates for each cPBMC (*n* = 3) and each WB (*n* = 3) specimen. As expected [30], the median CV was lower for abundant populations (i.e., CD3+ cells within lymphocytes, and CD4+ or CD8+ cells within CD3+ T cells), and higher for less-represented and poorly resolved subsets, such as CD4+ TD cells. In particular, acceptable precision values (CV below 0.20) were obtained for 8/15 vs. 12/15 parameters in cPBMC and for 6/15 vs. 13/15 parameters in WB experiments, in local vs. centralised analysis, respectively.

Regarding bias, mean values below 0.20 (considered acceptable) were achieved for 9/15 vs. 13/15 in cPBMCs and 7/15 vs. 14/15 parameters in WB locally vs. centrally analysed samples (Figure 5(b)).

Agreement among observations produced by operators when evaluating each cell subset was expressed as ICC, a statistical method independent of population size that estimates the ratio of biological to total variability [26, 27] (Figure 5(c)). For comparisons between groups, ICCs of 0.6–0.8 are considered adequate [31], while they should be >0.9 when taking patient-specific decisions [32]. Here we assume that ICC values above 0.75 were acceptable, as reported by other authors [26]. ICC laid above the acceptability threshold for 5/15 vs. 7/15 in cPBMC and for 2/15 vs. 11/15 parameters in WB samples, in local vs. centralised analysis.

We can point out that some subpopulations were subject to more variability in cPBMC as compared to WB setting. Specifically, the CD8-N subset proved to be out of the acceptability range for both accuracy (CV and Bias) and agreement (ICC), thus turning out to be the less reliable parameter under study. Other cPBMC subsets sensitive to variability were CD3-EM (high CV value), CD3-TD, CD3-EM, CD8-CM, and CD3-CM (low ICC value), as well. Finally, CD4+ cells were prone to low interoperator agreement, even though they are a well-represented and identifiable subset; this finding might be mainly ascribable to the cryopreservation handling which contributes to the interassay uncertainty to interdonor variability.

CD4-TD suffered from poor accuracy but displayed acceptable agreement among operators in both WB and cPBMC settings. On the other hand, in WB samples, CD4-EM showed low reproducibility (low CV), while CD8-EM and all of the CM subsets (CD3-, CD4-, and CD8-) displayed low ICC.

Based on previously described operator's performance results (Supplementary Fig. [Supplementary-material supplementary-material-1] and Figure 4), we recalculated the agreement by the ICC method on WB centrally analysed data, selecting operators with *Z*-score values between -1.5 and 1.5 in Figure 4, thus excluding operators B, F, H, and K. This selection improved operator concordance for most parameters, even if 2 out of 15 (CD3-CM and CD4-CM) were still below 0.75 thresholds (Supplementary Fig. [Supplementary-material supplementary-material-1]).

### 3.5. Multivariate Analysis

**Figure 6:**
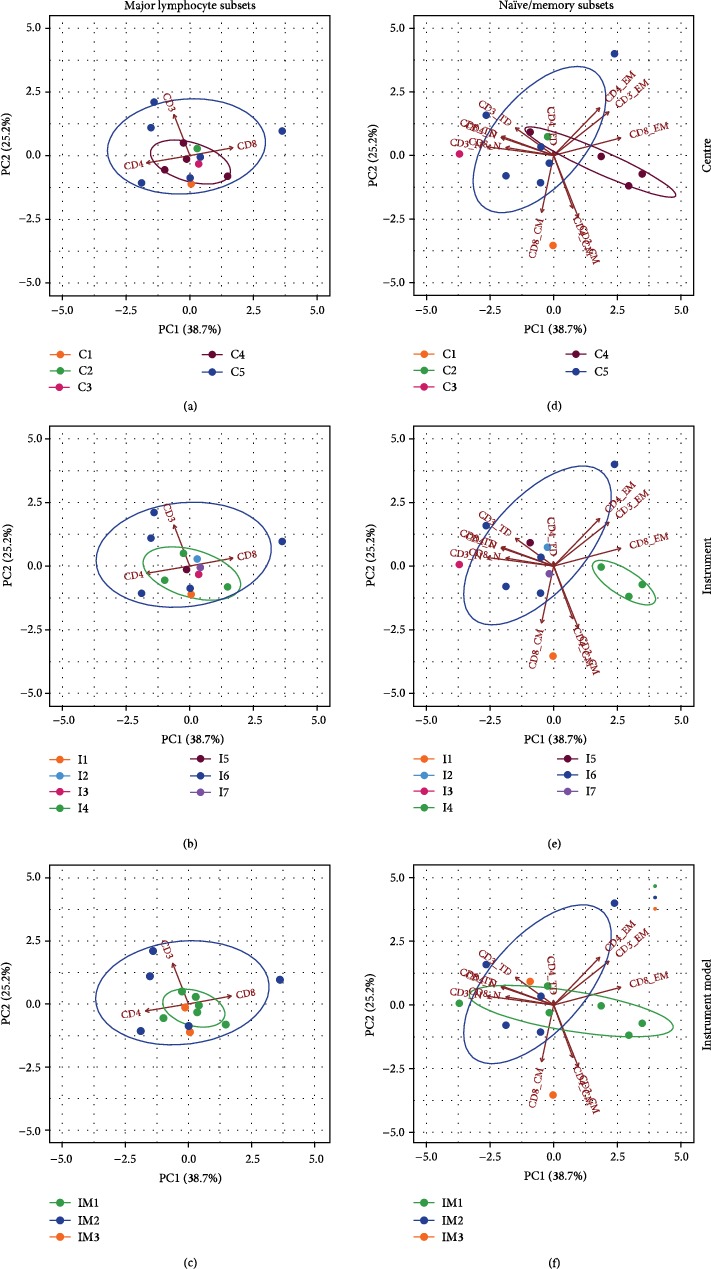
Multivariate analysis by PCA. Biplots displaying both operators' readings (points) and parameters (vectors). Confidence ellipses are provided (CI = 95%) for operators' readings after grouping them according to “Centre” (a, d), specific “Instrument” (b, e), and “Instrument model” (c, f) to analyse the impact of such factors on major (a–c) and naïve/memory (d–f) lymphocyte subsets. Ellipses can be drawn when at least 3 readings are available in groups. Results from one representative WB donor are shown.

Principal component analysis (PCA) was used to explore whether observations of multiple populations clustered according to three possible sources of variability: centre, specific instrument, and instrument model. To this aim, PCA was applied on WB centrally analysed data only, to exclude variability due to local gating and compensation as well as the interassay uncertainty of the cPBMC model (Figure 6).

In WB, the PCA analysis revealed that in parent populations (CD3+, CD4+, and CD8+; Figures 6(a)–6(c)), the first two components accounted for 99.4% of total variance. CD4 and CD8 almost entirely contribute to the first principal component and were anticorrelated, as expected; the second principal component primarily accounted for CD3 variability. After clustering, data related to centre no. 5 (C5 in Figure 6(d)) appeared more dispersed as compared to other centres. Similarly, instrument no. 6 (I6 in Figure 6(e)) and instrument model no. 2 (IM2 in Figure 6(f)) showed the poorest performance. As a consequence, it was not feasible to uniquely identify a possible source of variability among those evaluated for major lymphocyte subpopulation.

On the other hand, PCA of naïve/memory cell subsets (Figures 6(d)–6(f)) revealed that 63.9% of the variance was represented in a 2-dimensional space. As a general rule, the variables were grouped according to the naïve/memory subpopulation type rather than original (parent) population. EM variables mostly contributed to the first component and were negatively correlated to TD and N variables. CM variables contributed mostly to the second component. Operator observations showed to be grouped separately along the first principal component according to the instrument used for the acquisition, but neither to the centre operator's work in nor to the instrument model. In particular, instrument no. 4 (I4 in Figure 6(e), the green ellipse) is apparently prone to an EM variable overestimation. Nevertheless, data obtained by two out of three operators using this instrument did not show a higher variability compared to others, so that we had no reasonable argument to exclude instrument no. 4 from global analysis.

### 3.6. Instrument Sensitivity

To address instrument no. 4 behaviour, we compared each parameter fluorescence intensity profiles derived from WB central analysis (Supplementary Fig. [Supplementary-material supplementary-material-1]), within the indicated parent population, selecting data produced by the most performant operator for each instrument (as per Figure 4). As expected [33], CCR7 and CD45RA profiles were characterised by a typical pattern, with negativity and positivity peaks not neatly separated; therefore, discrimination between positive and negative events could be easily biased. It is worth noting that CCR7 was poorly resolved (2 channel decades of fluorescence intensity by instrument no. 4 vs. 3 channel decades by most of the instruments), causing greater difficulty in distinguishing positive from negative events. No differences among instruments were found for the comparing resolution index [21] relative to each marker of the panel (data not shown).

Graphical study of bidimensional plots, built on the CCR7/CD45RA expression in CD3+ cells, confirmed that poorly resolved parameters could generate difficult positioning of negativity/positivity quadrants (Supplementary Fig. [Supplementary-material supplementary-material-1]).

## 4. Discussion

Multicolour flow cytometry is a powerful tool for phenotypical and functional characterisation of immune cell subpopulations; however, the use of this technique in multicentre clinical trials has historically been limited by complexity, costs, and inconsistent methods for sample handling, reagents, instrument set-up, and data analysis among laboratories. So far, many international initiatives have supported programs for the harmonisation of flow cytometry methods [11–13, 34].

Here, we present a FCM harmonisation workflow directed by ISS among laboratories belonging to the immunomonitoring working group of the Lazio regional network for translational medicine, based on a previously described “mixed model” [35], to simulate a real multicentre study, where an operator's individual capacity in sample handling, processing, and analysis by his/her own instrumentation and software are maintained. This approach primarily constitutes a concrete tool to evaluate reproducibility of data as well as to identify and stem obstacles related to critical elements affecting variability in FCM assays.

Although in recent years a considerable amount of complex panels with an increasing number of parameters has been developed, we defined a basic panel, compliant with equipment of involved laboratories, identifying circulating naïve/memory T lymphocytes that represent clinically relevant cellular populations in different cancer immunotherapy settings [18, 19].

As a versatile tool to be used in both cPBMC and WB settings, we designed a backbone reagent consisting of a dried mixture of five antibodies essential to detect naïve/memory lymphocyte subsets.

Similar dried antibody mixtures have already proven to yield high-data reproducibility and reliability in large-scale projects such as the ONE study [13], the PreciseADS study [34], and others [35–37]. These reagents contain exactly the same amount of dried antibodies precoated in individual tubes for direct labelling of cells from the same batch and are very stable overtime, offering the advantage of speeding up and simplifying the labelling procedure, reducing the number of technical steps, and avoiding the maximum of biases.

Despite computational automated methods having reached a sufficient level of maturity and accuracy for reliable use in flow cytometry, in this study, we focused on manual analysis, since most centres are not equipped with bioinformatics resources and expertise. It is well known that manual gating [38] is a critical issue in FCM immunomonitoring assays. For this reason, we wanted to estimate to what extent analysis centralisation could amend local variability. To this aim, raw acquisition files were centrally reanalysed by an expert reference operator at ISS (ROP), who showed *ex post* one of the best accuracy score among participants.

We finally generated two independent data sets for cPBMC and WB samples, considering that both biological matrices might offer advantages and disadvantages in the context of clinical studies.

In this regard, the use of cPBMCs facilitates management and shipment costs, enabling simultaneous retrospective analysis of samples, collected from the same patient at different time points. However, PBMC isolation and cell freezing are not always feasible in all centres. In addition cryopreservation procedures as well as the use 24-hour-old buffy coat might lead to some artefacts and importantly modify the *ex vivo* immune cell composition [26, 39, 40], even though a resting time lapse might restore frequencies of some cell populations [41]. In fact, when taking into account central analysed data only, cPBMCs showed a TD cell increase as well as alterations of other subset frequencies [39, 40].

On the other hand, fresh whole blood does not require complex preanalytic phases and more accurately reflects the *ex vivo* cellular composition, enabling the detection of granulocytes otherwise excluded by the Ficoll density separation.

Despite a preliminary stability test having confirmed that delayed staining and acquisition of WB samples may generate artefacts in some population frequency evaluation [12], we set up WB staining 24 hours after blood withdrawn and sample acquisition on the following day. This procedure allowed simulating a realistic sample management in a multicentre study [42, 43] and also fulfilled specific needs of some involved laboratories. Several reports used blood stabilisers in order to delay sample staining and acquisition, with different outcomes depending on analysed markers [44]. However, we considered these reagents not suitable for interassay variability testing of naïve/memory subpopulations since, in our experience, CCR7 can be downregulated.

Concerning operator performance, in locally analysed cPBMCs, each participant displayed an adequate concordance with reference median values while, for some of them, precision values were found out of acceptability range in naïve/memory subsets and analysis centralisation could just mitigate but not abolish this trend, as also observed in interoperator analysis.

In WB, some operators displayed high repeatability but failed to analyse samples in terms of concordance for many parameters, including CD3, CD4, and CD8. This variability might be ascribable most likely to improper individual interpretation of the SOP relative to T cell gating, probably due to the fact that WB is less employed for immunophenotyping among participating laboratories with respect to PBMC samples.

Regarding subset reliability, data variability in both cellular models was very limited for large lymphocyte subpopulations as expected, while it was wider for some naïve/memory cell subsets. In fact, these populations are defined by the bidimensional expression of CCR7 and CD45RA, two markers characterised by continuous and poorly resolvable expression profiles, which require to be processed by high-resolution cytometers. Distinctively, CD4-TD cells showed the worst CV and bias values both in the interoperator study of variability and in the intraoperator analysis of precision, while their agreement among operators was acceptable in both cPBMC and WB settings. These findings were not surprising since CD4-TD showed very low percentages (nearby zero) and CV and bias are influenced by the arithmetic mean, while ICC index is not. For this reason, when evaluating a FCM assay variability, we recommend to calculate all of the described indexes and parameters, since they can reflect different features related to it.

In the WB setting, most participants revealed good trueness and good agreement. Nevertheless, some corrective measures are still required to reach a complete control of reproducibility for our panel, since few memory subsets persisted out of the acceptability range even after selecting the best performant operator centrally analysed data.

Overall results confirmed that WB should be preferred to cPBMCs if centrally analysed, for two main reasons: (i) it is less affected by variability [13] and (ii) it more truthfully reflects *ex vivo* peripheral leukocyte composition [41]. However, we should remind that we cannot make direct comparison, because distinct donors were used in the two settings and because we are dealing with interassay replicates for cPBMC and intra-assay replicates for WB.

In addition, our data demonstrated that the major impact of variability is due to operator intervention, while we had evidence that instrument models and respective configurations did not influence variability in a great extent. Thus, we confirmed that in studies involving multiple laboratories, it would be practicable to operate on different brands [45] and types of cytometers, as long as they are maintained under high-quality standard to avoid misleading results.

## 5. Conclusion

The present study shows that our approach based on shared protocols, centralised FCM data analysis, and extensive statistical analysis allows to identify variability sources and to achieve well-harmonised and reproducible results in the WB setting, overcoming assay variability among operators. We highlighted that the main factors affecting variability are represented by the individual conformity to SOPs related to sample handling and gating procedures. In fact, precise gating is a key prerequisite of reliable data and centralisation of FCM analysis enabled to reach reproducible results, thus allowing to bypass local analysis variability. Moreover, some corrective measures, such as more detailed SOPs for PBMC thawing and WB red blood lysing procedures, are still required in order to optimise reproducibility and consistency among different laboratories. Improved procedures are needed as well to detect improper sensitivity among different cytometers [46].

Finally, our results demonstrated that the proposed panel is suitable for immunomonitoring of peripheral blood naïve/memory T cells in multicentre clinical trials, provided that instruments employed pass the proper quality check and operators are well trained and coordinated.

In general, a tuning phase, by means of a proficiency testing approach, should be carried out before starting any multicentre immunomonitoring among participating laboratories. Controlling and correcting for technical variability will enable the dissection of true biological variation among subjects, thus allowing the identification of possible immune biomarkers, either prognostic or predictive of response to therapy, that will help personalised therapeutic choice [47].

## Figures and Tables

**Table 1:** Participants, instruments, and software. Five centres, with a total of 13 operators (including the reference operator, ROP), using seven different flow cytometers dedicated to research use (except GMP-maintained BD FACSCanto™ I at ISS, with a fluidic system upgrade, comparable to a BD FACSCanto™ II), participated to the harmonisation panel. Three flow cytometer models with compatible optical configuration (BC Gallios™, BD FACSCanto™ II, and BD LSRFortessa™) were used. The data generated were analysed by operators at peripheral sites (local analysis) using their own analysis software (Kaluza, FlowJo, or FACSDiva). Central analysis at ISS was performed by the ROP with Kaluza software on local raw data (acquired fcs files).

5 centres	13 operators	7 instruments	3 instrument models	2 acquisition software	3 analysis software
ISS SUR INMI IRE OPBG	12 + 1 ROP	1 FACSCanto I+ (GMP) 3 BD FACSCanto II 2 BD LSRFortessa 1 BC Gallios	BD FACSCanto II BD LSRFortessa BC Gallios	BC Kaluza BD FACSDiva	BC Kaluza BD Diva TreeStar FlowJo

**Table 2:** Antibody specifications–naïve/memory panel. Antibody-fluorochrome conjugates for naïve/memory T cell phenotype panel. The panel was composed of a backbone dried mixture of five antibodies (a+b) and the drop-in liquid markers: live/dead discriminator for PBMC (a) and anti-CD45 APC-Cy7 for WB (b) samples.

	Marker	Fluorochrome	Clone	Sample	Supplier	Format
a+b	CD4	FITC	13B8.2	PBMC/WB	Beckman Coulter	DURAClone custom backbone (dried)
	CCR7 (CD197)	PE	G043H7	PBMC/WB		
	CD8	PeCy5.5	B9.11	PBMC/WB		
	CD3	PeCy7	UCHT-1	PBMC/WB		
	CD45RA	APC	2H4	PBMC/WB		

a	Dead exclusion marker	Near-IR (NiR)	Live/dead Fixable dead cell stain kit	PBMCs	Thermo Fisher	Drop-in (liquid)

b	CD45	APC-Cy7	2D1	WB	BioLegend	Drop-in (liquid)

## Data Availability

The datasets generated during and/or analysed during the current study are available on a web public repository specialized in Flow Cytometry data (http://www.flowrepository.org), identified by FlowRepository ID FR-FCM-Z282.

## Supplementary Materials

Supplementary Materials Supplementary Figure S1: (a) recovery and (b) viability of PBMCs after thawing. Data are shown for each of the three aliquots of each donor (PBMC1, PBMC2, and PBMC3) thawed in 3 different rounds by each operator (Op_A to Op_M). (a) The percentage of cells recovered regardless of viability (cell count after thawing/cell count before freezing); (b) the percentage of viable cells recovered (live cells/total counted cells after thawing) by each operator. Individual and median operator CVs are shown for both recovery and viability indices. Supplementary Figure S2: intraoperator variability. Interassay repeatability of each operator for each cell subset for PBMCs after local and central analysis ((a) and (b), respectively). In this case, CV was first calculated for each donor-specific triplicate (3 rounds) and then the median value was determined on the 3 donors. Intra-assay repeatability of each operator for each parameter for WB samples after local (c) or centralised (d) analysis. Here, the CV was calculated on the three experimental replicas (1 round), and then the median on the 3 donors was calculated. Centralisation mitigated interassay variability for some cPBMC data, while it did not affect WB intra-assay variability, which was already excellent in the local analysis. Supplementary Figure S3: agreement among selected best performant operators. ICC values of WB centrally analysed data obtained excluding 4 operators that showed *Z*-score above 1.5 or below -1.5 in Figure 4(d). Supplementary Figure S4: fluorescence comparison among cytometers (1 Gallios Beckman Coulter, 4 BD FACSCanto, and 2 BD LSRFortessa). Data are shown from a representative WB sample. Analysis was performed at the central site using Kaluza software. Data are represented as fluorescence histograms for each parameter within the gated cells (CD45 within the singlet gate, CD3+ cells within the lymphocyte gate, and CD4+, CD8+ CD45RA, and CCR7+ cells within CD3+ gated cells). Supplementary Figure S5: CCR7-CD45RA bidimensional plots. Comparison among operators. Data are shown from a representative WB sample. Analysis was performed at the central site using Kaluza software. Data are represented as fluorescence plots within CD3+ cells. Supplementary Table S1: data report form. Participants were asked to fill the present form with their local analysis results for (a) cPBMCs and (b) WB samples. Survey questionnaire. Standard operating procedures (SOPs): (i) PBMC isolation and freezing; (ii) PBMC thawing and counting; (iii) PBMC staining, acquisition, and analysis; and (iv) WB staining, acquisition, and analysis.
