# Corrigendum to “Multicentre Harmonisation of a Six-Colour Flow Cytometry Panel for Naïve/Memory T Cell Immunomonitoring”

**DOI:** 10.1155/2020/2698258

**Published:** 2020-08-10

**Authors:** Iole Macchia, Valentina La Sorsa, Irene Ruspantini, Massimo Sanchez, Valentina Tirelli, Maria Carollo, Giorgio Fedele, Pasqualina Leone, Giovanna Schiavoni, Carla Buccione, Paola Rizza, Paola Nisticò, Belinda Palermo, Stefania Morrone, Helena Stabile, Aurelia Rughetti, Marianna Nuti, Ilaria Grazia Zizzari, Cinzia Fionda, Roberta Maggio, Cristina Capuano, Concetta Quintarelli, Matilde Sinibaldi, Chiara Agrati, Rita Casetti, Andrea Rozo Gonzalez, Floriana Iacobone, Angela Gismondi, Filippo Belardelli, Mauro Biffoni, Francesca Urbani

**Affiliations:** ^1^Department of Oncology and Molecular Medicine, Istituto Superiore di Sanità (ISS), Rome 00161, Italy; ^2^Research Coordination and Support Service, CoRI, ISS, Rome 00161, Italy; ^3^Core Facilities, ISS, Rome 00161, Italy; ^4^Department of Infectious Diseases, ISS, Rome 00161, Italy; ^5^Center for Gender-Specific Medicine, ISS, Rome 00161, Italy; ^6^Unit of Tumor Immunology and Immunotherapy, IRCCS Regina Elena National Cancer Institute (IRE), Rome 00128, Italy; ^7^Department of Experimental Medicine, Sapienza University of Rome (SUR), 00185, Italy; ^8^Department of Molecular Medicine, SUR, Rome 00185, Italy; ^9^Clinical Research, Imperial College, London W12 ONN, UK; ^10^Onco-Hematology Department, IRCCS Bambino Gesù Children's Hospital (OPBG), Rome 00165, Italy; ^11^Cellular Immunology Laboratory, IRCCS National Institute for Infectious Diseases “L. Spallanzani” (INMI), Rome 00149, Italy; ^12^Institute of Translational Pharmacology, National Research Council, Rome 00185, Italy; ^13^Medical Biotechnology and Translational Medicine PhD School, Tor Vergata University, Rome 00133, Italy

In the article titled “Multicentre Harmonisation of a Six-Colour Flow Cytometry Panel for Naïve/Memory T Cell Immunomonitoring” [1], there was an error in Table 1. The table should show affiliation of one of the two BD LSR Fortessa instruments used in the study and cited in Table 1. The corrected table is shown below and is listed as Table 1.

## Figures and Tables

**Table 1:** Participants, instruments, and software. Five centres, with a total of 13 operators (including the reference operator, ROP), using seven different flow cytometers dedicated to research use (except GMP-maintained BD FACSCanto™ I at ISS, with a fluidic system upgrade, comparable to a BD FACSCanto™ II), participated to the harmonisation panel. Three flow cytometer models with compatible optical configuration (BC Gallios™, BD FACSCanto™ II, and BD LSR Fortessa™) were used. The data generated were analysed by operators at peripheral sites (local analysis) using their own analysis software (Kaluza, FlowJo, or FACSDiva). Central analysis at ISS was performed by the ROP with Kaluza software on local raw data (acquired fcs files). ^∗^One of the two BD LSR Fortessa instruments, used by one operator from SUR center, belongs to the core facilities of the CLNS: Center for Life NanoScience@Sapienza, Istituto Italiano di Tecnologia, Rome, Italy.

5 centres	13 operators	7 instruments	3 instrument models	2 acquisition software	3 analysis software
ISS SUR INMI IRE^∗^ OPBG	12+1 ROP	1 FACSCanto I+ (GMP) 3 BD FACSCanto II 2 BD LSRFortessa^∗^ 1 BC Gallios	BD FACSCanto II BD LSRFortessa BC Gallios	BC Kaluza BD FACSDiva	BC Kaluza BD Diva TreeStar FlowJo

## References

[1] Macchia I., la Sorsa V., Ruspantini I. (2020). Multicentre harmonisation of a six-colour flow cytometry panel for naïve/memory T cell immunomonitoring. *Journal of Immunology Research*.

