# Supplementary material for: Multicentre Harmonisation of a Six-Colour Flow Cytometry Panel for Naïve/Memory T Cell Immunomonitoring
Source: J Immunol Res. 2020 Apr 12;2020:1938704. doi: 10.1155/2020/1938704 (PMC7153001; doi:10.1155/2020/1938704)
Supplement: Supplementary Materials — Supplementary Figure S1: (a) recovery and (b) viability of PBMCs after thawing. Data are shown for each of the three aliquots of each donor (PBMC1, PBMC2, and PBMC3) thawed in 3 different rounds by each operator (Op_A to Op_M). (a) The percentage of cells recovered regardless of viability (cell count after thawing/cell count before freezing); (b) the percentage of viable cells recovered (live cells/total counted cells after thawing) by each operator. Individual and median operator CVs are shown for both recovery and viability indices. Supplementary Figure S2: intraoperator variability. Interassay repeatability of each operator for each cell subset for PBMCs after local and central analysis ((a) and (b), respectively). In this case, CV was first calculated for each donor-specific triplicate (3 rounds) and then the median value was determined on the 3 donors. Intra-assay repeatability of each operator for each parameter for WB samples after local (c) or centralised (d) analysis. Here, the CV was calculated on the three experimental replicas (1 round), and then the median on the 3 donors was calculated. Centralisation mitigated interassay variability for some cPBMC data, while it did not affect WB intra-assay variability, which was already excellent in the local analysis. Supplementary Figure S3: agreement among selected best performant operators. ICC values of WB centrally analysed data obtained excluding 4 operators that showed Z-score above 1.5 or below -1.5 in Figure 4(d). Supplementary Figure S4: fluorescence comparison among cytometers (1 Gallios Beckman Coulter, 4 BD FACSCanto, and 2 BD LSRFortessa). Data are shown from a representative WB sample. Analysis was performed at the central site using Kaluza software. Data are represented as fluorescence histograms for each parameter within the gated cells (CD45 within the singlet gate, CD3+ cells within the lymphocyte gate, and CD4+, CD8+ CD45RA, and CCR7+ cells within CD3+ gated cells). Supplementary Figure S5: CCR7 [file 1938704.f1.pdf]

Supplementary figures and tables

Multicentre harmonisation of a six-colour flow cytometry panel for naïve/memory T cell immunomonitoring

Iole Macchia[1*], Valentina La Sorsa[2], Irene Ruspantini[4], Massimo Sanchez[4], Valentina Tirelli[4], Maria Carollo[4], Giorgio Fedele[5], Pasqualina Leone[5], Giovanna Schiavoni[1], Carla Buccione[1], Paola Rizza[3], Paola Nisticò[6], Belinda Palermo[6], Stefania Morrone[7], Helena Stabile[8], Aurelia Rughetti[7], Marianna Nuti[7], Ilaria Grazia Zizzari[7], Cinzia Fionda[8], Roberta Maggio[9], Cristina Capuano[7], Concetta Quintarelli[10], Matilde Sinibaldi[10], Chiara Agrati[11], Rita Casetti[11], Andrea Rozo Gonzalez[1], Floriana Iacobone[1], Angela Gismondi[8], Filippo Belardelli[1,12], Mauro Biffoni[1], Francesca Urbani[1,13*].

Affiliations:

1. Department of Oncology and Molecular Medicine, Istituto Superiore di Sanità (ISS), Rome, Italy
2. *Research* Coordination and *Support Service - CoRI* , ISS, Rome, Italy
3. Center for Gender-specific Medicine, ISS, Rome, Italy
4. Core Facilities- Cytometry Unit, ISS, Rome, Italy
5. Department of Infectious Diseases, ISS, Rome, Italy
6. Unit of Tumor Immunology and Immunotherapy - IRCCS Regina Elena National Cancer Institute (IRE), Rome, Italy
7. Department of Experimental Medicine, Sapienza University of Rome (SUR), Italy
8. Department of Molecular Medicine, SUR, Rome, Italy
9. Clinical Cancer Research, Imperial College, UK
10. Onco-Hematology Department IRCCS Bambino Gesù Children's Hospital (OPBG), Rome, Italy
11. Cellular Immunology Laboratory, National Institute for Infectious Diseases "L. Spallanzani" (INMI), Rome, Italy
12. Institute of Translational Pharmacology, National Research Council, Rome, Italy
13. Medical Biotechnology and Translational Medicine PhD School, Tor Vergata University - Rome, Italy

[*] *Correspondence and requests for materials should be addressed to: iole.macchia@iss.it; francesca.urbani@iss.it*

Summary

1. Supplementary Figures and Tables
2. Survey Questionnaire
3. Standard Operating Procedures (SOPs):
   - PBMC isolation and freezing
   - PBMCs thawing and counting
   - PBMC staining, acquisition and analysis
   - WB staining, acquisition and analysis

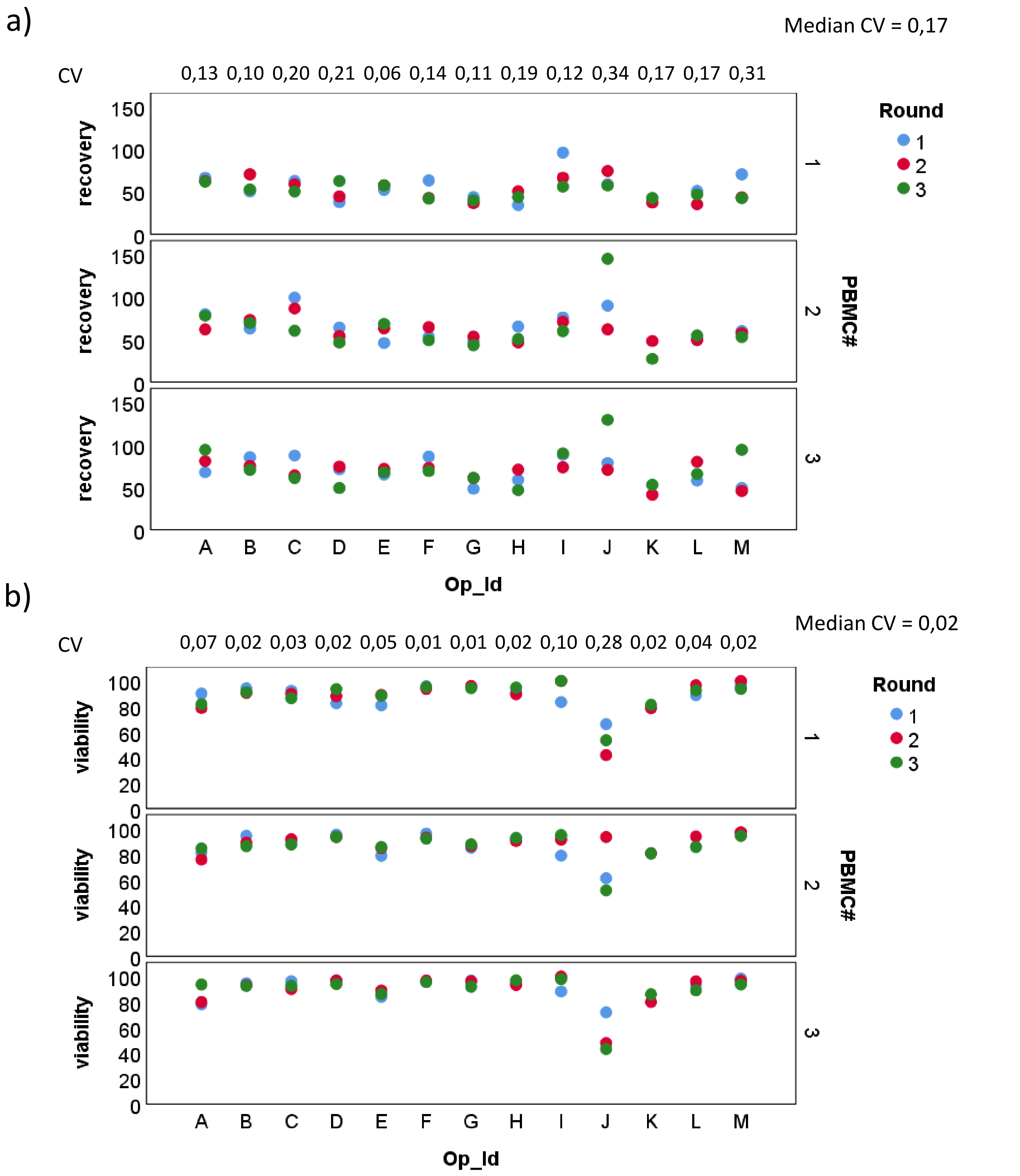

Supplementary Figure S1. a) recovery and b) viability of PBMCs after thawing. Data are shown for each of the three aliquots of each donor (PBMC1, PBMC2, PBMC3) thawed in 3 different rounds by each operator (Op_A to Op_M). a) the percentage of cells recovered regardless of viability (cell count after thawing/cell count before freezing); b) the percentage of viable cells recovered (live cells/ total counted cells after thawing) by each operator. Individual and median operator CV are shown for both recovery and viability index.

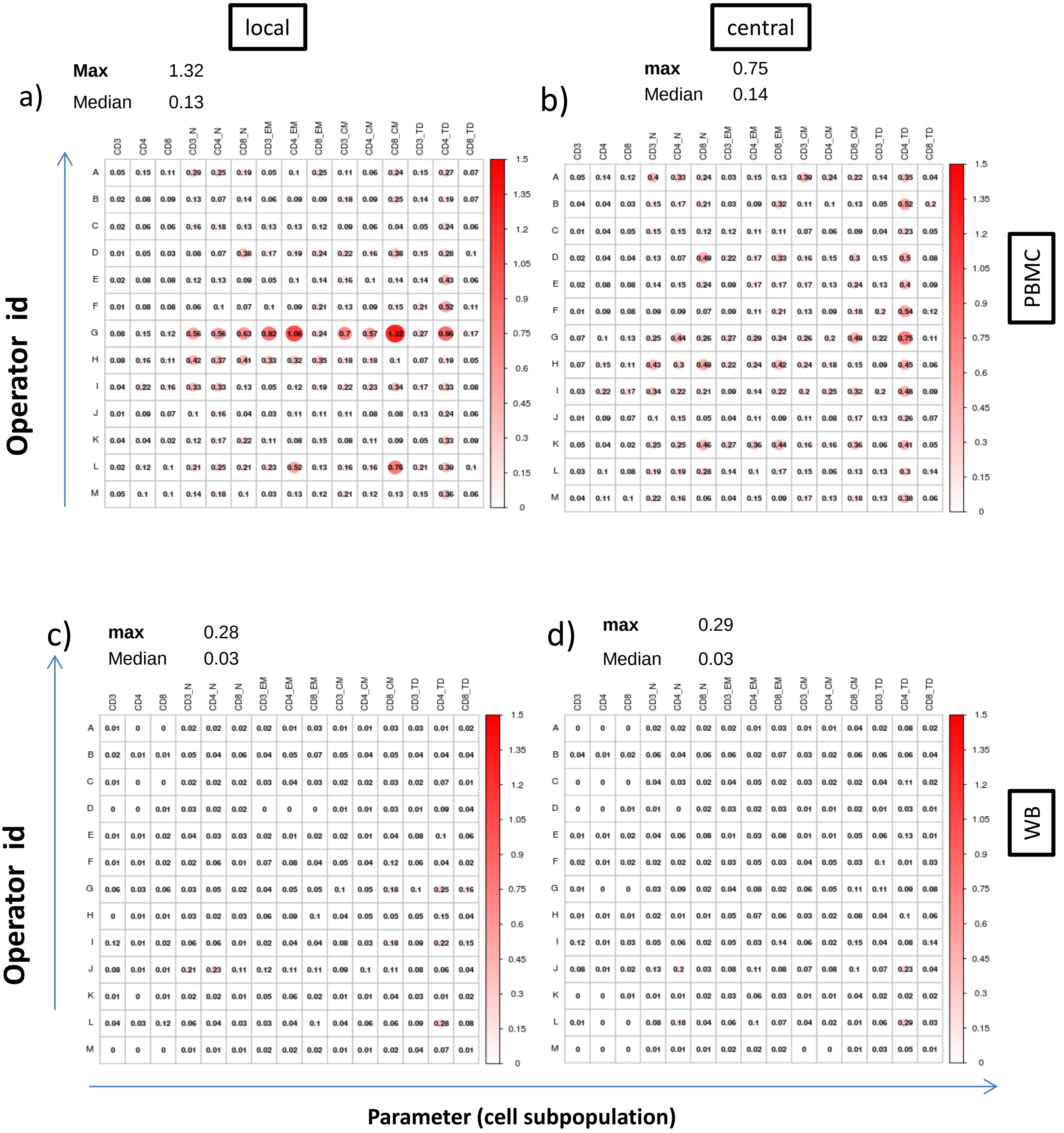

Supplementary Figure S2. Intra-operator variability. Inter-assay repeatability of each operator for each cell subset for PBMCs after local and central anaysis (a and b respectively). In this case CV was first calculated for each donor-specific triplicate (3 rounds) and then the median value was determined on the 3 donors.

Intra-assay repeatability of each operator for each parameter for WB samples after local (c) or centralized (d) analysis. Here the CV was calculated on the three experimental replicas (1 round) and then calculating the median on the 3 donors.

Centralisation mitigated inter-assay variability for some cPBMC data, while it did not affect WB intra-assay variability, which was already excellent in local analysis.

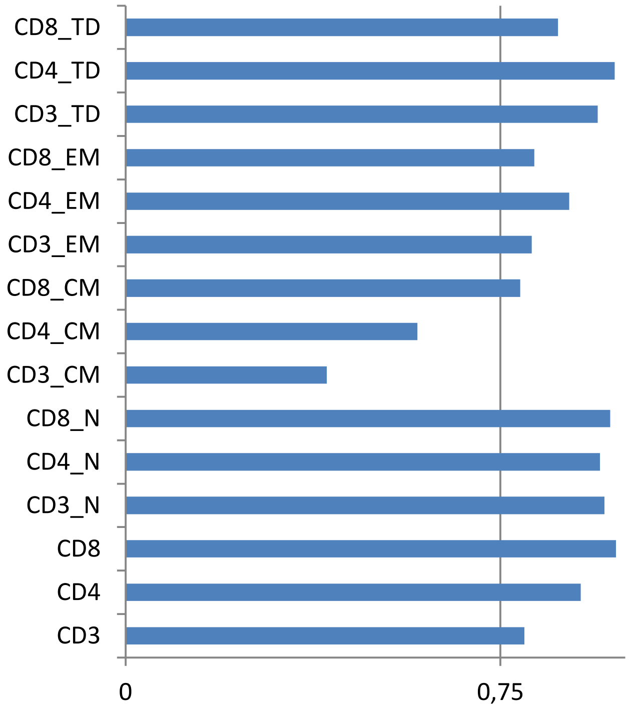

Supplementary Figure S3 . Agreement among selected best performant operators. ICC values of WB centrally analysed data obtained excluding 4 operators that showed Z-score above 1,5 or below -1,5 in Figure 4d.

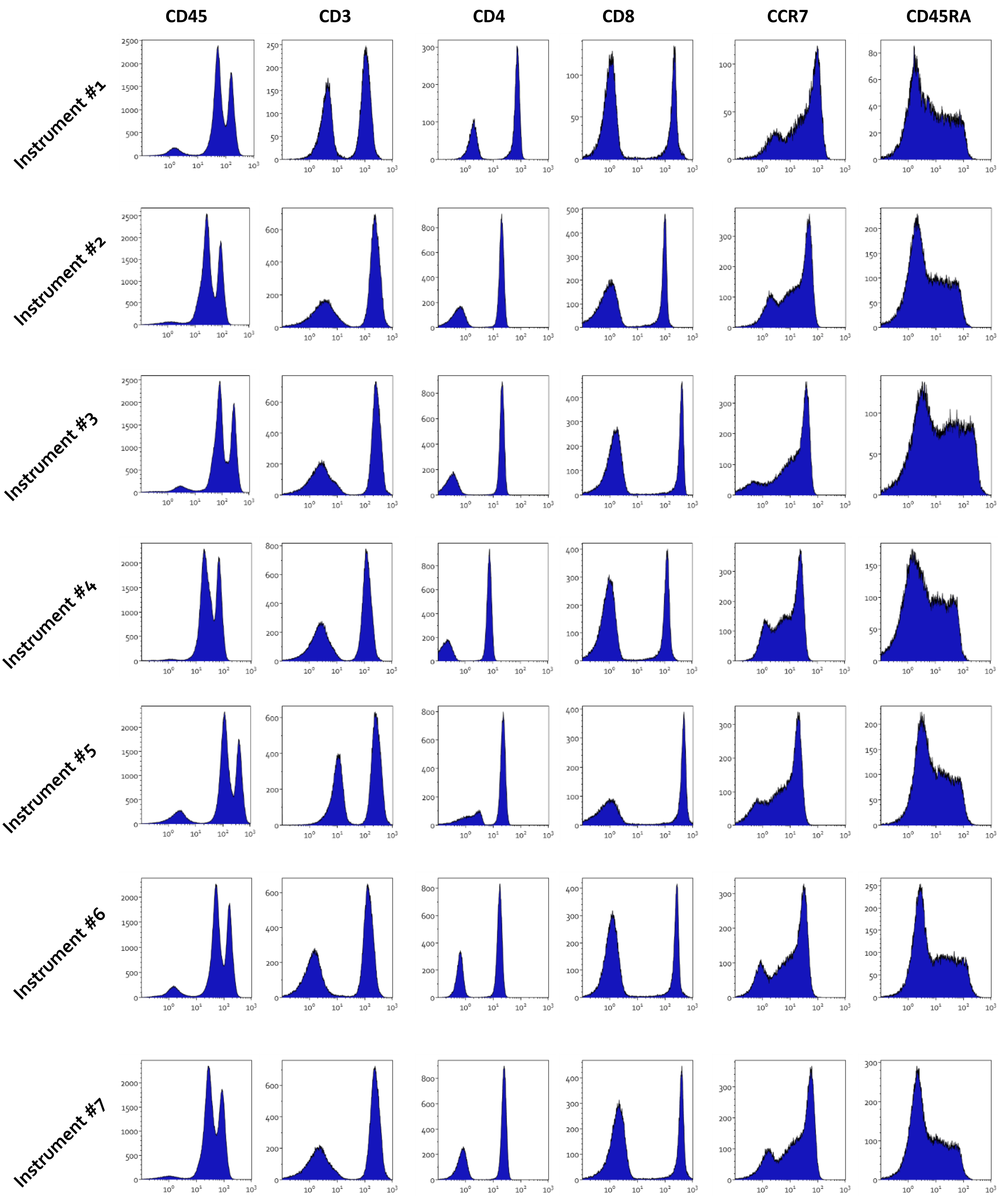

Supplementary Figure S4. Fluorescence comparison among cytometers (1 Gallios Beckman Coulter, 4 BD FACS Canto and 2 BD LRS Fortessa). Data are shown from a representative WB sample. Analysis was performed at central site using Kaluza software. Data are represented as fluorescence histograms for each parameter within the gated cells (CD45 within singlets gate, CD3+ cells within Lymphocyte gate, CD4+, CD8+ CD45RA, CCR7+ cells within CD3+ gated cells.

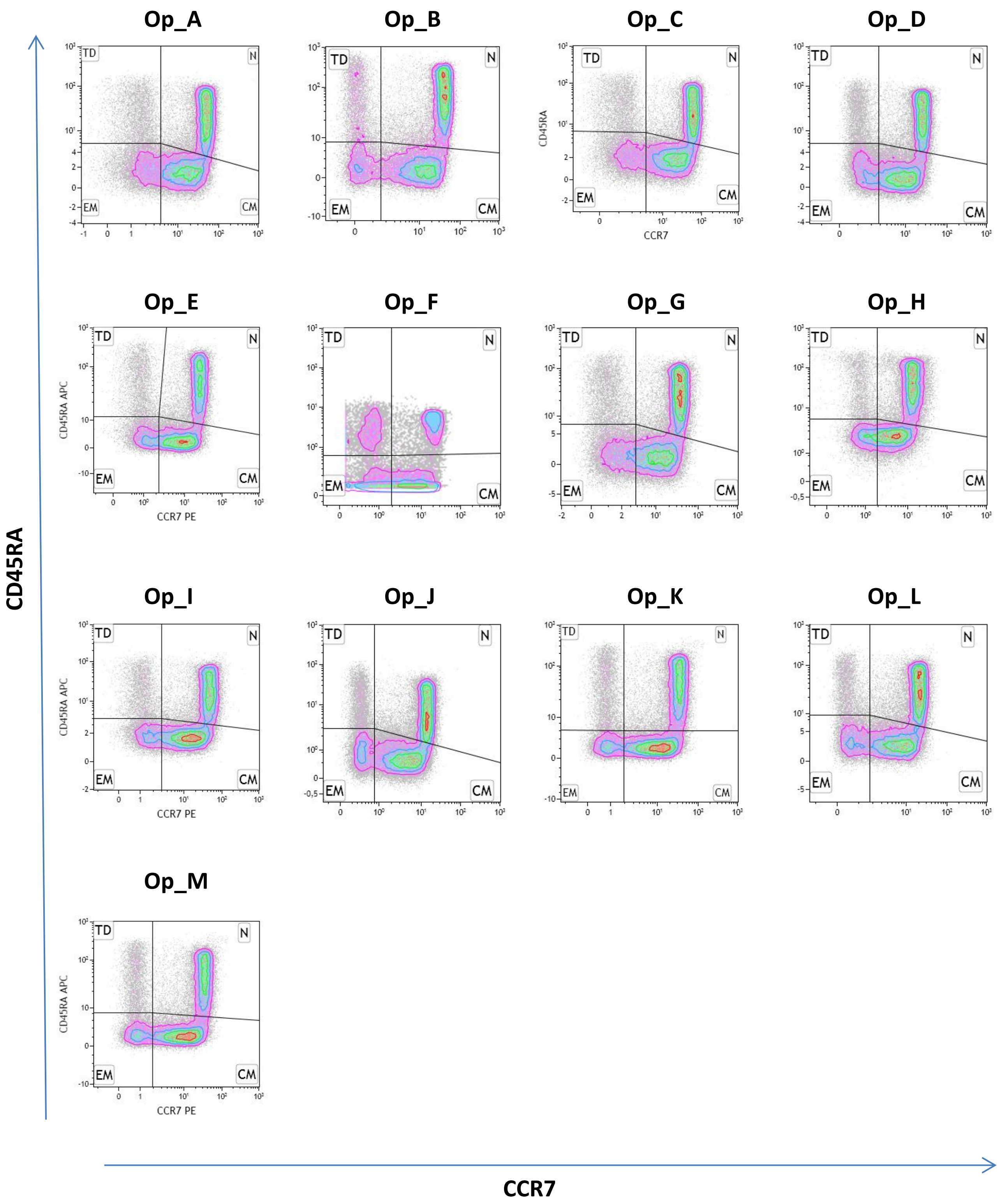

Supplementary Figure S5. CCR7-CD45RA bi-dimensional plots. Comparison among operators. Data are shown from a representative WB sample. Analysis was performed at central site using Kaluza software. Data are represented as fluorescence plots within CD3+ cells.

Date<br>Centre (IRE, OPBG, INMI, SUR, ISS)<br>Operator ID (Op_A-Op_M)<br>File name (Op_Id_ WB ##_round #)<br>FCM instrument model<br>Analysis software<br>Notes of acquisition and analysis (any anomaly or artefact)<br>Number of total events			
**a) cPBMCs**		**b) WB**	
Round (1-2-3). Different days<br>Vial (PBMC1, PBMC2, PBMC3)<br>live cells after thawing (milions)<br>dead cells after thawing (milions)		Replicate (1-2-3)- same day<br>Vial (WB1,WB2, WB 3)<br>stained volume (µl)	
**Parent population**	**% of gated**	**Parent population**	**% of gated**
All events	Time ok	All events	Time ok
Time ok	Live cells	Time ok	Singlets
Live cells	Singlets	Singlets	Leucocytes (CD45+)
Singlets	Lymphocytes	Leucocytes (CD45+)	Lymphocytes
Lymphocytes	CD3	Lymphocytes	CD3
CD3+	CD3 CM	CD3+	CD3 CM
	CD3 EM		CD3 EM
	CD3 N		CD3 N
	CD3 TD		CD3 TD
CD3+	CD4	CD3+	CD4
CD4+	CD4 CM	CD4+	CD4 CM
	CD4 EM		CD4 EM
	CD4 N		CD4 N
	CD4 TD		CD4 TD
CD3+	CD8	CD3+	CD8
CD8+	CD8 CM	CD8+	CD8 CM
	CD8 EM		CD8 EM
	CD8 N		CD8 N
	CD8 TD		CD8 TD

Supplementary Table S1. Data Report form. Participants were asked to fill the present form with their local analysis results for: a) cPBMCs and b) WB samples.

Lazio Region Project

Survey questionnaire of Lazio region Immunomonitoring Centres

Authors: Iole Macchia, Francesca Urbani – Istituto Superiore di Sanità (ISS)

Date: 2016-12-01

Revised by: Valentina La Sorsa on 2019-07-05

Summary

Reference Centre contacts 2
Affiliation institute 2
General information of the centre 3
Quality standard 3
Immunoassays 4
Molecular assays 6
Biostatistics and bioinformatics 6

Reference Centre contacts (* mandatory field)

**Name of the centre***	
**Acronym of the centre**	
**Address of the centre***	
**Head of the centre***	
**Website**	
**E-mail***	
**Phone***	
**Fax**	
**Legal status**	
**Other**	

Affiliation institute

Type of institution*	▪ Hospital<br>▪ University<br>▪ Public institute<br>▪ Private institute<br>▪ IRCSS<br>▪ Company<br>▪ Public-private consortium<br>▪ Association of patients<br>▪ Other (specify)
Denomination*	
Address (only if different from the centre address)	
Legal representative	
Scientific director	
Website	
E-mail	
Telephone and fax numbers	

General information

Mission of the Centre*	☐ Research<br>☐ Therapy<br>☐ Services<br>☐ Other (specify)
Areas of interest*	☐ Immuno-oncology<br>☐ Other (specify)
Facilities/Expertise present in the Institution/Centre	☐ Flow cytometry<br>☐ Cellular imaging<br>☐ Molecular imaging<br>☐ Genomics<br>☐ Transcriptomics<br>☐ Proteomics<br>☐ Systems biology<br>☐ Other (specify)

Quality standard

Quality assessment	☐ External accreditation programs (UK NEQAS - EQA)<br>☐ Participation in proficiency panels<br>☐ Internal method validation and standardization<br>☐ SOP system
Good Laboratory Practice (GLP) Compliance	(yes/no)
Laboratory organization: Adequate rooms with separate areas, sample storage areas, controlled environmental conditions	(yes/no)
Instrumentation and equipment: calibrated and periodically maintained instruments, log-book presence	(yes/no)
Staff: Qualified personnel, adequate number of personnel. Presence of documentation attesting qualification and training	(yes/no)
Number of people working in the centre	unit of personnel

Materials and reagents: properly labeled materials, expiration control system	(yes/no)

Immunoassays

Pathology under study, specify*	
Type of immune response under study*	☐ innate/adaptive ☐ humoral/cellular
Sample type	☐ Peripheral blood<br>☐ Biopsy fragment<br>☐ Lymph nodes<br>☐ Bone marrow<br>☐ Fine needle biopsies<br>☐ Other (specify)
Cellular assays for phenotypic and functional cell analysis	☐ Lymphoproliferation assays (thymidine incorporation ^{3}H, BrdU, CFSE, MTT, other - specify)<br>☐ Cytotoxicity assays (release of ^{51}Cr, CD107 degranulation assay, other- specify)<br>☐ Flow cytometry (FCM) based assays for multiparametric analysis of cellular phenotype<br>☐ Cellular imaging<br>☐ Generation and maintenance of Ag-specific cell lines and clones<br>☐ Other (specify)
Cytokine and chemokine dosage	☐ ELISA (Enzyme-Linked Immunosorbent Assay)<br>☐ ELISPOT (Enzyme-Linked ImmunoSpot)<br>☐ FluoroSpot (Fluorescence-based dual-colour enzyme-linked immunospot)<br>☐ ICS (intracellular cytokine staining)- FCM analysis<br>☐ Multiplex Arrays (specify)<br>☐ CBA (BD Cytometric Bead Array)<br>Other (specify)

Antigen specific cell assay	☐ Frequency analysis of antigen-specific T lymphocytes<br>☐ Characterization of antigen-specific T lymphocytes<br>☐ Isolation of antigen-specific T lymphocytes<br>☐ In situ labeling of antigen-specific T lymphocytes<br>☐ Multimers (HLA tetramers, pentamers, dextramers) staining (specify)<br>Other (specify)
Circulating antibody assays	☐ Titration<br>☐ Isotyping<br>☐ Immune precipitation<br>☐ Agglutination assay<br>☐ Complement fixing<br>☐ Immune fluorescence microscopy<br>☐ ELISA<br>☐ SDS-PAGE electrophoresis<br>☐ RIA, "Radio Immuno-Assay"<br>Other (specify)

TCR Molecular analysis	☐ TCR Spectratyping<br>☐ RNA seq<br>Other (specify)

Specify the type of instrumentation used for each assay.

Instrumentation	

Flow Cytometry	☐ Flow cytometer analyzers: (Gallios (Beckman Coulter) ,LSR-Fortessa x20 (Becton Dickinson-BD), FACSCanto (BD), Other (specify)<br>☐ Sorter: FacsAria III (Becton Dickinson), MoFlo Astrios EQ (Beckman Coulter), Other<br>Number of available fluorescences<br>Acquisition / analysis programs FlowJo, Kaluza, Summit, AutoGate, Diva7 and Diva8, etc. (specify)<br>Programs for computational analysis of flow cytometric data / data mining software (SPICE, other) (specify)

Molecular assays

- ☐ Southern blot
- ☐ Sequencing (Southern, SOLEXA, SNP ecc)
- ☐ NGS
- ☐ PCR and Real time PCR
- ☐ Classical cytogenetics (karyotyping)
- ☐ FISH (Fluorescence *in situ* hybridization)
- ☐ CGH-array (molecular karyotype)
- ☐ Northern blot
- ☐ Study of miRNA
- ☐ Microarray
- ☐ RT-PCR e Real time RT- PCR
- ☐ Western blot
- ☐ Mass spectrometry

Other (specify)

Biostatistics and bioinformatics

Specific database for immunological and molecular monitoring data presence	(yes/no)
Data management system	(yes/no)
eCRF	(yes/no)
Other electronic patient data recording	(yes/no)
Software (R, IBM-SPSS, STATA, SAS, Other - specify)	

Standard Operating Procedure (SOP): "Human PBMCs Isolation and Freezing from buffy coat"

Authors: Iole Macchia, Francesca Urbani – Istituto Superiore di Sanità (ISS)

Date: 2017-09-01

Revised by: Valentina La Sorsa on 2019-07-05

Warning: This protocol provides for the use of human blood derivatives. Operators should have received an appropriate training and they must work according to laboratory safety guidelines for hemoderivative products.

Purpose: This document describes the process for isolating and freezing stocks of human peripheral blood mononuclear cell (PBMC) samples from healthy donor blood bank buffy coat, to be used in the procedure described in SOP "PBMC thawing and counting .pdf" file.

Experimental plan

Procedure will be performed at ISS, main center of the harmonisation project, on 24-hour buffy coats released by the Policlinico Umberto I Transfusion Center, Rome, Italy. <u>Gradient separation and freezing at -80°C must be performed consecutively, without pauses, on the same day. Liquid nitrogen freezing will be executed at least after 6 hours (even the day after).</u>

Table 1. Reagents

Ethanol 70°	Sigma Aldrich, Zwijndrecht, The Netherlands
Heparin 4000 UI/ml (Clexane)	Sanofi, Paris, France
LymphoPrep™ Solution	Axis-Shield PoC AS, Oslo, Norway
Phosphate Buffered Saline, w/o Ca and Mg (PBS)	Lonza, MD, USA
FBS, heat inactivated fetal bovine serum	Euroclone, Pero Milan, Italy; heat-inactivated 30' at 56°C
Human Serum Albumin (HSA) 20% Solution for Infusion	Baxter AG, Vienna, Austria,
Trypan Blue 0.4% solution	To be diluted 1:4 with 2D H_2O and filtered before use.
Dimethyl Sulfoxide (DMSO)	Sigma Aldrich, Zwijndrecht, The Netherlands
Freezing medium	10% DMSO, 5% HSA, 85% PBS;store at at +4°C

Table 2. Materials and Equipment

Class A biological safety cabinet	
Centrifuge	To be used at 350-800 g (g to rpm online conversion tool: http://www.endmemo.com/bio/grpm.php)

Vortex	
Thermostatic water bath	
50 ml sterile conical tubes	
1-2 ml vial	
Pipette Aid and sterile disposable pipettes	
Micropipette Set and tips	
Sterile scissors	
Neubauer type cell counting chamber	After use, it should be left at least 20' in a benzalconium chloride solution at 2%, then rinsed with distilled water and degreased with ethanol 70°
Cryovials	
Isopropanol freezing cryobox	Check isopropanol to be at appropriate level and cool it at +4°C before use.

Method

PBMCs Isolation

Preliminary operations

1. Use 3 sets of 5 x 50 ml conical tube for each buffy coat bag, one for PBS dilution, one for Lymphoprep gradient and one for PBMC ring collection.
2. Label the conical tubes for each sample (PBMC1, PBMC2 and PBMC3).
3. Record sex, date of birth, date of withdrawal, bag identification code (or bar code).
4. Warm Lymphoprep and PBS at RT before use.

For each buffy coat sample proceed as follows:

5. Thoroughly disinfect one of the exit tubes of the buffy coat bag with Ethanol 70°.
6. Cut it with sterile scissors.
7. Let the blood to flow into a conic 50 ml vial.
8. Squeeze the bag to recover as much blood volume as possible.
9. Record the collected blood volume (usually 50-60 ml).
10. Add 1 µl of heparin for each ml of buffy coat blood.
11. Mix blood and heparin by sterile pipetting.
12. Dispense 10 ml of blood into each of the 5 falcon conical tubes (first set).
13. Dilute 1:3 blood, by adding 20 ml PBS to each first set tube.
14. Dispense 20 ml of Lymphoprep into the second set of tubes.
15. Pipette up and down to homogenize the blood with the PBS solution in each first set tube.
16. Carefully stratify the diluted blood onto the Lymphoprep contained in the second set tubes.
17. Centrifuge at 21°C, 800 g, mild acceleration, no brake, for 30’.
18. For each centrifuged tube, discard most of the plasma/PBS above the PBMC ring (approximately 1 cm above the white ring).
19. Collect the PBMC ring by a 5ml pipette.
20. Transfer each ring into a new tube (third set) and fill it in with PBS, up to 50 ml.
21. Centrifuge at 21°C, 400 g, max acceleration, max brake, 10’.
22. Discard the supernatant.
23. Resuspend the pelleted cells in 5ml of PBS.
24. Pipette up and down and reunite cellular suspension from all tubes in a single one.
25. Adjust volume to 50 ml with PBS.
26. Centrifuge at 21°C, 350 g, max acceleration, max brake, 10’.

27. Discard the supernatant.
28. Resuspend in 10 ml of PBS.

Cell counting:

29. Dispense in a 1-2 ml vial 980 μl of Trypan Blue.
30. Add 20 μl of cellular suspension (dilution 1:100).
31. Vortex for 5''.
32. Collect 10 μl and place them on a Neubauer-type chamber.
33. Count both live and dead cells by using a 10x objective lens in an inverted microscope in 2 opposite quadrants of the chamber (Fig. 1). The amount of live cells must be between 35 and 100 in each quadrant: if this does not happen, repeat by correcting the dilution factor.

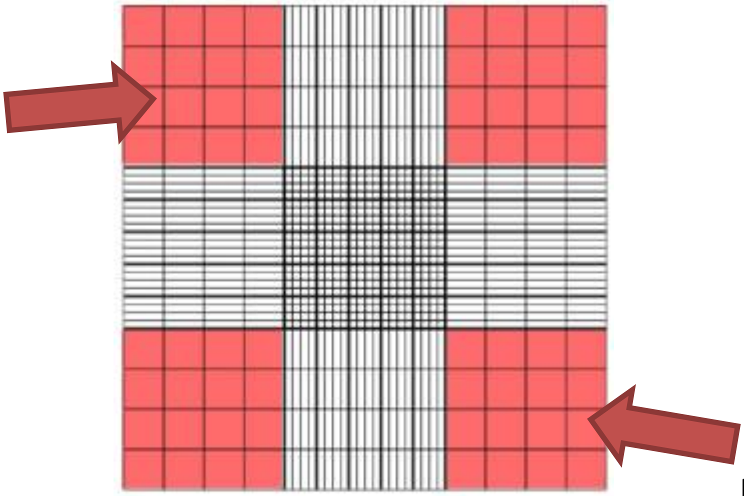

Figure 1

34. Calculate the number of PBMCs /ml (= “mean cell number of a 4x4 quadrant” x “10^4“ x “dilution factor”).
35. Record the information on a worksheet, according to the scheme (example):

Dilution factor	Number of live cells 1st quadrant	Number of live cells 2nd quadrant	Number of quadrants counted	Cell suspension Volume (ml)	10^6/ml
100	45	55	2	1	50,00

Dilution factor	Number of dead cells 1st quadrant	Number of dead cells 2nd quadrant	Number of quadrants counted	Cell suspension Volume (ml)	10^6/ml
100	4	5	2	1	4,5

PBMCs freezing

36. Centrifuge at 21°C, 350 g, max acceleration, max brake, 10’.
37. Discard the supernatant.
38. Resuspend cells in an appropriate volume of freezing medium in order to dispense 5-8 x 10^6 cells/0.5 ml/cryovial.
39. Dispense 0.5 ml per cryovial and put them in the cryobox.
40. Place the cryobox at - 80°C for at least 6h (ON), then transfer cryovials to liquid nitrogen until use.

Standard Operating Procedure (SOP):
"Thawing and counting cryopreserved human PBMCs"

Authors: Iole Macchia, Francesca Urbani – Istituto Superiore di Sanità (ISS)

Date: 2017-09-01

Revised by: Valentina La Sorsa on 2019-07-05

Warning: This protocol provides for the use of human blood derivatives. Operators should have received an appropriate training and they must work according to laboratory safety guidelines for hemoderivative products.

Purpose: This document describes the process for the thawing of peripheral blood mononuclear cell (PBMC) samples to be used in the SOP described in "PBMC staining acquisition and analysis.pdf" file. It would be executed by all operators belonging to the harmonisation project.

Table 1. Reagents

Foetal Bovine Serum	Own, heat-inactivated 30' at 56°C
Phosphate Buffered Saline, w/o Ca and Mg (PBS)	Own
Trypan-blue	Own, filter it before using
DNAse Sigma cod#S4881- 5 mg/ml (250x)	Supplied by ISS
Thawing buffer A	PBS 20% FBS, supplemented with DNAse. Use 25 ml per vial to be thawed. For example, for 3 vials add 300 μl of DNAse 250 x to 75 ml of PBS containing 15 ml of FBS just before use. To be prepared just before use.
Thawing buffer B	PBS 10% FBS, supplemented with DNAse. Use 25 ml per vial to be thawed. For example, for 3 vials add 300 microliters of DNAse 250 x to 75 ml of PBS containing 7.5 ml of FBS just before use. To be prepared just before use.

Table 2. Materials and Equipment (Own)

Class A biological safety cabinet	
Centrifuge	To be used at 200-500 g (online conversion tool from g to rpm: http://www.endmemo.com/bio/grpm.php)
Vortex	
Micropipette Set	
Pipet Aid	
Water bath	
50 ml Sterile, Polypropylene, Conical, Centrifuge Tubes	
Sterile pipets	

Before the procedure:

- Turn on the thermostatic bath at 37 ° C.
- Warm up solution at room temperature.

Method:

1. Thaw quickly and incompletely by warming the cryovials at 37°C in the water bath.
2. Add 1 ml of thawing buffer A to the cryovial.
3. Pipette gently and slowly transfer cell suspension into a 50 ml conical tube, containing 25 ml of thawing buffer A.
4. Fill up the 50 ml conical tube with thawing buffer A.
5. Centrifuge at 350 g for 5 minutes at RT.
6. Decant the supernatant by inversion.
7. Resuspend the pellet in 25 ml of thawing buffer B.
8. Centrifuge at 350 g for 5 minutes at room temperature.
9. Resuspend the pellet in 1 ml of PBS.
10. Vortex for a few seconds.
11. Gently mix the cell suspension by pipetting to remove any clumps of cells. Mix 20µl of cell suspension and with 180 µl of 0.4% Trypan blue in an empty vial (1:10 dilution).
12. Vortex 5’’.
13. Pipette 10 µl of the cell/Trypan blue mixture on a Neubauer counting chamber.
14. Count both live and dead cells by using a 10x objective lens in an inverted microscope.
15. Count both live and dead cells by using a 10x objective lens in an inverted microscope in 2 opposite quadrants of the chamber (Fig. 1). The amount of live cells must be between 35 and 100 in each quadrant: if this does not happen, repeat by correcting the dilution factor.

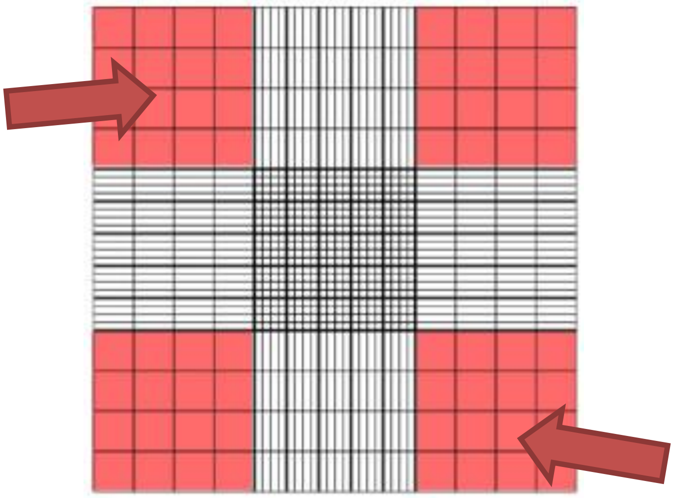

16. Calculate number of PBMCs /ml (= “mean cell number of a 4x4 quadrant” x “10^4“ x “dilution factor”).
17. Record the information on a worksheet, according to the scheme (example):

**Dilution factor**	**Number of live cells 1st quadrant**	**Number of live cells 2nd quadrant**	**Number of quadrants counted**	**Cell suspension Volume (ml)**	**10^6/ml**
10	45	55	2	1	5,00

**Dilution factor**	**Number of dead cells 1st quadrant**	**Number of dead cells 2nd quadrant**	**Number of quadrants counted**	**Cell suspension Volume (ml)**	**10^6/ml**
10	4	5	2	1	0,45

18. Go to SOP “PBMC staining acquisition and analysis.pdf”.

SOP: "Staining, acquisition and analysis of human cPBMCs with a 6 colour flow cytometry panel for naïve/memory T cell detection"

Authors: Iole Macchia, Francesca Urbani – Istituto Superiore di Sanità (ISS)

Date: 2017-09-01

Revised by: Valentina La Sorsa (ISS) on 2019-07-05

Warning: This protocol provides for the use of human blood derivatives. Operators should have received an appropriate training and they must work according to laboratory safety guidelines for hemoderivative products.

Purpose: This document describes the process for staining, acquisition and analysis of thawed human PBMC samples with a 6 colour flow cytometry panel for naïve/memory T cell detection. PBMC thawing procedure is described in "PBMC thawing and counting.pdf" file. It would be executed by all operators belonging to the harmonisation project.

Experimental plan

For each round, the entire procedure (thawing, staining and acquisition) must be performed consecutively, without pauses, on the same day.

Each operator will test 1 PBMC sample for each of the 3 donors, in 3 experimental rounds to obtain 3 replicas from each donor. The entire procedure will be carried out over a period of about twenty days, with a lapse of 6- 7 days between one session and another, within the month of October 2017.

First round is described in section a); second and third rounds in section b).

Table 1. Composition of the 6 colour panel: Duraclone Custom integrated with a live/dead cell discriminator

	Marker	Fluorochrome	Clone
Beckman Coulter (BC) Duraclone Custom, dried	CD4	FITC	13B8,2
	CCR7 (CD197)	PE	G043H7
	CD8	PE Cy5.5	B9.11
	CD3	PE Cy7	UCHT-1
	CD45RA	APC	2H4
ThermoFisher, liquid	LIVE/DEAD™ Fixable Near-IR Dead Cell Stain Kit,	NIR	

Table 2. Reagents

Duraclone tubes (BC # B38658, custom product)	Supplied by Istituto Superiore di Sanità, 5-colour dried tubes.
Duraclone custom tubes BC # B38658 - Compensation kit	Supplied by Istituto Superiore di Sanità, contains single-colour dried tubes.
LIVE/DEAD™ Fixable Near-IR Dead Cell Stain Kit, ThermoFisher	Supplied by Istituto Superiore di Sanità.
Phosphate Buffered Saline, w/o Ca and Mg (PBS)	Own, any brand
Staining solution	Own, 1X PBS, 2% FBS, 2mM EDTA, Sodium Azide 0.09%
Formaldehyde	Own, 0.8% solution in PBS

Table 3. Materials and Equipment (Own)

Class A biological safety cabinet	
Centrifuge	To be used at 350 g (g to rpm online conversion tool): http://www.endmemo.com/bio/grpm.php)
Vortex	
Micropipette Set and tips	
Flow cytometry Tubes	
Flow Cytometer	At least 2 lasers: 488 and 630/640 nm. It must undergo an internal quality control of alignment (required), sensitivity and linearity (highly recommended).

Table 4. Tube labeling.

Type	Label	Tube	round 1	round 2	round 3
**6-colour tubes**	PBMC1/6 colour	1 empty + 15-colour Duraclone tube	X	X	X
	PBMC2/6 colour	1 empty + 15-colour Duraclone tube	X	X	X
	PBMC3/6 colour	1 empty + 15-colour Duraclone tube	X	X	X
**Unstained ctr**	PBMC2/UNST	1 empty tube	X		
**Duraclone single colour compensation tubes**	PBMC2/NIR	1 empty tube	X		
	PBMC2/FITC	1 comp. kit tube (CD4 FITC)	X		
	PBMC2/PE	1 comp. kit tube (CCR7 PE)	X		
	PBMC2/PeCy5.5	1 comp. kit tube (CD8 PeCy5.5)	X		
	PBMC2/PeCy7	1 comp. kit tube (CD3 PeCy7)	X		
	PBMC2/APC	1 comp. kit tube (CD45RA APC)	X		

a) Method for the first experimental round (includes compensation)

Thawing and counting of the three donor cPBMCs (PBMC1, PBMC2 and PBMC3), is described in the SOP provided by Istituto Superiore di Sanità (thawing and counting.pdf).

Staining

1. Centrifuge the 3 cPBMC vials (350 g, 5’, RT) and discard the supernatant.
2. Resuspend the pellet in staining solution at the concentration of $1x10^6$ cells/ml.
3. Label tubes according to table 4.
4. Aliquot 1 ml ($1x10^6$) of each cPBMC vial into the 3 empty tubes labeled as PBMC1, PBMC2, PBMC3 and into the tubes identified as PBMC2/UNST and PBMC2/NIR.
5. Keep residual cells of the donor PBMC2 to + 4 ° C, to be used for the compensation step (see point 13).
6. Add 1µl of NIR into the 3 tubes labeled PBMC1, PBMC2, PBMC3 and into the PBMC2/NIR tube (refreeze NIR at -20 ° C for subsequent rounds).
7. Vortex and incubate for 20’ at RT in a dark place.
8. Add 2 ml of staining solution, centrifuge at 350 g for 5 'at RT and discard the supernatant.
9. Add 2 ml of staining solution and vortex.
10. Centrifuge at 350 g for 5' at RT and aspirate the supernatant.
11. For tubes PBMC1, PBMC2, PBMC3: Resuspend the pellet in 100 µl of staining solution and transfer the cells in the respective "Duraclone Custom" tubes previously labeled as PBMC1, PBMC2, PBMC3.
12. For PBMC2/UNST and PBMC2/NIR tubes: resuspend the pellet in 100 µl of staining solution.
13. Dispense 0.5 ml (0.5×10^6) of PBMC2 donor into each of the five single-colour Duraclone "compensation kit" tubes.
14. Vortex all tubes (Duraclone Custom, compensation tubes, PBMC2/UNST and PBMC2/NIR) and incubate 15 'at RT in the dark.
15. Add 2 ml of PBS 1X to all tubes, vortex, centrifuge at 350 g for 5 'at RT and discard the supernatant.
16. Resuspend in 150 µl of Formaldehyde 0.8%. Incubate at 4 ° C in a dark place for at least 3’.
17. Just before the acquisition, add 150 µl of 1X PBS and acquire samples in the flow cytometer within 3 hours as reported by Kalina T. et al. (Leukemia, 2012 Sep; 26 (9): 1986-2010).

Acquisition

1. Acquire the compensation kit tubes, the PBMC2/NIR tube and the PBMC2/UNST tube and perform your own compensation routine.
2. For each 6-colour Duraclone tube, acquire 200,000 events in ALL (PBMC1, PBMC2 and PBMC3), recording the 6 Fluorescence, FS-H, FS-A, SS-A and TIME channels. Use the compensation matrix generated in point 1 making manual adjustment, if necessary. Name the file as: Operator_Id _ round#_PBMC# (example OpA_R1_PBMC1).
3. Send the acquired ".fcs" files via email to francesca.urbani@iss.it and iole.macchia@iss.it .

b) Method for the second and third experimental rounds

Thawing and counting of the three donor cPBMCs (PBMC1, PBMC2 and PBMC3), is described in the SOP provided by Istituto Superiore di Sanità (thawing and counting.pdf).

Staining

1. Centrifuge the 3 cPBMC vials (350 g, 5’, RT) and discard the supernatant.
2. Label tubes according to table 4.
3. Centrifuge at 350 g for 5 'at RT and discard the supernatant.
4. Resuspend the cells in staining solution at the concentration of $1x10^6$ cells/ml.

5. Aliquot 1 ml ($1x10^6$) of PBMC into the 3 empty tubes labeled as PBMC1, PBMC2, PBMC3.
6. Add 1µl of NIR in each tube.
7. Vortex and incubate for 20 minutes at RT in a dark place.
8. Add 2 ml of staining solution, centrifuge at 350 g for 5' at RT and discard the supernatant.
9. Add 2 ml of staining solution and vortex.
10. Centrifuge at 350 g for 5' at RT and aspirate the supernatant.
11. Resuspend the pellet in 100 µl of staining solution and transfer the cells into the previously labeled "Duraclone Custom" tubes (PBMC1, PBMC2, PBMC3).
12. Incubate 15' at RT in a dark place.
13. Add PBS 1X (2 ml/tube), vortex, centrifuge at 350 g for 5' at RT and discard the supernatant.
14. Resuspend in 150 µl of Formaldehyde 0.8%. Incubate at 4°C in dark place for at least 30'.
15. Just before acquisition, add 150 µl of 1X PBS and acquire samples by the flow cytometer within 3 hours.

Acquisition

1. Use the instrument setting and the compensation matrix of the first round.
2. For each 6-colour Duraclone tube, acquire 200,000 events in ALL (PBMC1, PBMC2 and PBMC3), recording the 6 Fluorescence, FS-H, FS-A, SS-A and TIME channels. Name the file as: Operator_Id _ round#_PBMC# (example OpA_R2_PBMC1).
3. Send the acquired ".fcs" files via email to francesca.urbani@iss.it and iole.macchia@iss.it .

Analysis and gating strategy

1. Perform analysis by using your current analysis software (BD Diva, TreeStar FlowJo, BC Kaluza or other).
2. Check compensation and perform adjustment with an off-line tool, if needed. (All files should be acquired with the same instrument settings and analysis should be performed with the same compensation matrix).
3. Adjust biexponential scale if your software allows it. Adjust each detector so that all populations are clearly defined and the negative populations are not pushed up against each axis.
4. For each 6 colour file, according to Figure 1, draw the following plots:
    - a TIME vs SS-A plot to select a stable acquisition time lapse by a gate (TIME OK).
    - Within TIME OK: a NIR vs FS-A plot to select live cells (negative for NIR).
    - Within the live cell gate: a FS-H vs FS-A plot to select singlet events.
    - Within the singlet event gate: a FS-A vs SS-A plot to identify the lymphocyte population.
    - Within the lymphocyte gate: a CD3 vs SS-A plot to identify CD3+ lymphocytes.
    - Within the CD3+ lymphocyte gate: a CD8 vs CD4 plot to identify the CD8 + and CD4 + single positive (SP) lymphocyte gates. Exclude double positive events from gating.
    - Separately, for each CD3+, CD4+ SP and CD8+ SP gates: a CD45RA vs CCR7 plot, to define the naïve/memory subpopulations (naïve - N: CD45RA + CCR7 +; central memory - CM: CD45RA-CCR7 +; effector memory - EM: CD45RA-CCR7-; terminally differentiated - TD: CD45RA + CCR7-).
5. Report the data obtained by your analysis in the “data report form”; name it as (report_PBMC_Op_Id.xlxs) and send it to francesca.urbani@iss.it and iole.macchia@iss.it .

Figure 1. Representative gate statistics for PBMCs (analysed by Kaluza software)

Gate	Number	%Gated
All	54.348	100,00
TIME OK	46.838	86,18
live cells	44.070	94,09
singlets	43.407	98,50
lymphocytes	33.964	78,25
CD3+	27.840	81,97
CD4 SP	11.713	42,07
CM_CD4	5.003	42,71
EM_CD4	5.119	43,70
N_CD4	1.364	11,65
TD_CD4	227	1,94
CD8 SP	14.915	53,57
CM_CD8	1.710	11,46
EM_CD8	2.466	16,53
N_CD8	1.091	7,31
TD_CD8	9.648	64,69
CM_CD3	6.978	25,06
EM_CD3	8.379	30,10
N_CD3	2.293	8,24
TD_CD3	10.190	36,60

Standard Operating Procedure (SOP): “Staining, acquisition and analysis of human whole blood samples with a 6-colour flow cytometry panel for naïve/memory T cell detection”

Authors: Iole Macchia, Francesca Urbani – Istituto Superiore di Sanità (ISS)

Date: 2018-04-01

Revised by: Valentina La Sorsa on 2019-07-05

Warning: This protocol provides for the use of human blood derivatives. Operators should have received an appropriate training and they must work according to laboratory safety guidelines for hemoderivative products.

Purpose: This document describes the process for staining, acquisition and analysis of fresh whole blood (WB) samples with a 6 colour flow cytometry panel for naïve/memory T cell detection. It would be executed by all operators belonging to the harmonisation project.

Experimental plan

The entire procedure (collection, staining and acquisition) must be performed in 3 consecutive days:

- Day 0: EDTA collection from 3 healthy donors (at ISS); distribution of the blood and reagent to the operators within 3 hours
- Day 1: staining
- Day 2: acquisition to the flow cytometer

Each operator will have to test an aliquot of whole blood for each of the 3 healthy donors, in a single experimental round, in triplicate (to obtain 3 experimental replicas from each donor).

Table 1. Composition of the 6 colour panel: Duraclone Custom integrated with a pan-leukocyte marker

	Marker	Fluorochrome	Clone
Beckman Coulter (BC) Duraclone Custom, dried	CD4	FITC	13B8,2
	CCR7 (CD197)	PE	G043H7
	CD8	PE Cy5.5	B9.11
	CD3	PE Cy7	UCHT-1
	CD45RA	APC	2H4
Biolegend, liquid	CD45	APC Cy7	2D1

Table 2. Reagents

Duraclone tubes (BC # B38658, custom product)	Supplied by Istituto Superiore di Sanità, 5-colour dried tubes.
Duraclone custom tubes BC # B38658 - Compensation kit	Supplied by Istituto Superiore di Sanità, contains single-colour tubes.
Anti-human CD45 APC Cy7, Biolegend	Supplied by Istituto Superiore di Sanità,.
Staining solution	1X PBS, 2% FBS, 2mM EDTA, Sodium Azide 0.09%
Phosphate Buffered Saline (w/o Ca and Mg) - PBS	Own, any brand
BD 10x Lysing solution	Supplied by Istituto Superiore di Sanità . To be diluted 1x with 2D H_2O just before use
Formaldehyde	0.8% solution in PBS

Table 3. Materials and Equipment

Class A biological safety cabinet	
Centrifuge	To be used at 350-500 g (g to rpm online conversion tool: http://www.endmemo.com/bio/grpm.php)
Vortex	
Micropipette Set and tips	
Cytometer Tubes	
Flow Cytometer	At least 2 lasers: 488 and 630/640 nm. It must undergo an internal quality control of alignment (required), sensitivity and linearity (highly recommended).

Table 4. Tube labeling

Type	Label	Tube	replica 1	replica 2	replica 3
**6-colour tubes**	WB1/6colour (a, b or c)	1 empty + 1 5-colour Duraclone tube	a	b	c
	WB2/6colour (a, b or c)	1 empty + 1 5-colour Duraclone tube	a	b	c
	WB3/6colour (a, b or c)	1 empty + 1 5-colour Duraclone tube	a	b	c
**Unstained ctr**	WB1/UNST	1 empty tube	X		
**Duraclone Single colour Compensation tubes**	WB1/APC Cy7	1 empty tube	X		
	WB1/FITC	1 comp. kit tube (CD4 FITC)	X		
	WB1/PE	1 comp. kit tube (CCR7 PE)	X		
	WB1/PeCy5.5	1 comp. kit tube (CD8 PeCy5.5)	X		
	WB1/PeCy7	1 comp. kit tube (CD3 PeCy7)	X		
	WB1/APC	1 comp. kit tube (CD45RA APC)	X		

Method (includes** compensation**)

Staining

Day 0 (04/17/2018):

1. Each operator will receive an aliquot of whole blood in anticoagulant tubes (EDTA) from 3 healthy donors (WB1, WB2, WB3): a 1.5 ml aliquot from the WB1 donor and a 0.8 ml aliquot from donors WB1 and WB2.
2. Leave the aliquots of whole blood received, in the dark at TA.

Day 1 (04/18/2018):

3. Label tubes according to table 4.
4. Add 200ul of donor WB1 blood into the unstained tube.
5. Add 200 µl of donor WB1 blood to the various compensation kit compensation tubes and to the tubes labeled WB1/UNST and WB1/APC Cy7.
6. Vortex all the tubes.
7. Incubate 15 'at RT in a dark place.
8. Add 3 ml of Lysing Solution BD 1X to the tubes.
9. Mix by inverting them 2/3 times and incubate 10' at RT in a dark place.
10. Centrifuge for 5' at 500g.
11. Wash by adding 3 ml of staining solution.
12. Vortex and centrifuge at 350 g for 5' at RT.
13. Discard the supernatant and suspend in 150 µl Formaldehyde 0.8%.
14. Incubate at 4°C in a dark place for at least 30'.
15. Add 150 µl of PBS 1X and leave in a dark place at +4°C.

Acquisition

Day 2 (04/19/2018)

1. Acquire the compensation kit tubes, the WB1/NIR tube and the WB1/UNST tube and perform your own compensation routine.
2. For each 6-colour Duraclone tube, acquire 200,000 ALL events at the flow cytometer, recording the 6 Fluorescence, FS-H, FS-A, SS-A and TIME channels.
3. Use the compensation matrix generated in point 1 making manual adjustment, if necessary. Name the file as: Operator_Id_WB#_replica# (example OpA _WB1_a).
4. Send the acquired ".fcs" files via email to francesca.urbani@iss.it and iole.macchia@iss.it .

Analysis and gating strategy

1. Perform analysis by using your current analysis software (BD Diva, TreeStar FlowJo, BC Kaluza or other).
2. Check compensation and perform adjustment with an off-line tool, if needed. (All files should be acquired with the same instrument settings and analysis should be performed with the same compensation matrix).
3. Adjust biexponential scale if your software allows it. Adjust each detector so that all populations are clearly defined and the negative populations are not pushed up against each axis.
4. For each 6 colours file, according to Figure 1, draw the following plots:
   - a TIME vs SS-A plot to select a stable acquisition time lapse by a gate (TIME OK).
   - Within TIME OK: a FS-H vs FS-A plot to select singlet events.
   - Within the singlet event gate: a CD45 vs FS-A plot to select leukocytes.

- Within the leukocyte gate: a FS-A vs SS-A plot to identify the lymphocyte population.
- Within the lymphocyte gate: a CD3 vs SS-A plot to identify CD3 + lymphocytes.
- Within the CD3 + lymphocyte gate: a CD8 vs CD4 plot to construct the two CD8 + and CD4 + single positive (SP) lymphocyte gates. Exclude double positive events from the gating.
- Separately, for each CD3+, CD4+ SP and CD8+ SP gates: a CD45RA vs CCR7 plot, to define the naïve/memory subpopulations (naïve - N: CD45RA + CCR7 +; central memory - CM: CD45RA-CCR7 +; effector memory - EM: CD45RA-CCR7-; terminally differentiated - TD: CD45RA + CCR7-).

5. Report the data obtained by your analysis in the "data report form"; name it as (report_WB_Op_Id.xlxs) and send it to francesca.urbani@iss.it and iole.macchia@iss.it .

Figure 1. Representative gate statistics for whole blood samples (analysed by Kaluza software)

Gate	Number	%Total	%Gated
All	54.629	100,00	100,00
TIME OK	50.182	91,86	91,86
singlets	47.376	86,72	94,41
Leuko	36.055	66,00	76,10
lymphocytes	17.580	32,18	48,76
CD3+	13.934	25,51	79,26
CD3_CM	4.196	7,68	30,11
CD3_EM	2.964	5,43	21,27
CD3_N	3.854	7,05	27,66
CD3_TD	2.920	5,35	20,96
CD4+	7.886	14,44	56,60
CD4_CM	3.853	7,05	48,86
CD4_EM	1.200	2,20	15,22
CD4_N	2.708	4,96	34,34
CD4_TD	125	0,23	1,59
CD8+	4.753	8,70	34,11
CD8_CM	435	0,80	9,15
CD8_EM	1.136	2,08	23,90
CD8_N	892	1,63	18,77
CD8_TD	2.290	4,19	48,18
